# [MIII2MII3]^*n*+^ trigonal bipyramidal cages based on diamagnetic and paramagnetic metalloligands†
†Electronic supplementary information (ESI) available: Additional NMR, IR, PXRD, ICPOES, MS, EPR data and CASSCF methodology. CCDC 1520425–1520429. Crystallographic data (including structure factors) for **1–5** have been deposited with the Cambridge Crystallographic Data Centre. For ESI and crystallographic data in CIF or other electronic format see DOI: 10.1039/c7sc00487g



**DOI:** 10.1039/c7sc00487g

**Published:** 2017-05-19

**Authors:** S. Sanz, H. M. O'Connor, V. Martí-Centelles, P. Comar, M. B. Pitak, S. J. Coles, G. Lorusso, E. Palacios, M. Evangelisti, A. Baldansuren, N. F. Chilton, H. Weihe, E. J. L. McInnes, P. J. Lusby, S. Piligkos, E. K. Brechin

**Affiliations:** a EaStCHEM School of Chemistry , The University of Edinburgh , David Brewster Road , Edinburgh , EH9 3FJ , UK . Email: E.Brechin@ed.ac.uk ; Email: S.Calvo@ed.ac.uk ; Email: Paul.Lusby@ed.ac.uk; b UK National Crystallography Service , Chemistry , University of Southampton , Highfield Campus , Southampton , SO17 1BJ , UK; c Instituto de Ciencia de Materiales de Aragón (ICMA) , CSIC – Universidad de Zaragoza , Departamento de Física de la Materia Condensada , 50009 Zaragoza , Spain; d School of Chemistry , The University of Manchester , Oxford Road , Manchester , M13 9PL , UK . Email: eric.mcinnes@manchester.ac.uk; e Department of Chemistry , University of Copenhagen , Universitetsparken 5 , DK-2100 , Copenhagen , Denmark . Email: piligkos@chem.ku.dk

## Abstract

A family of five [MIII2MII3]^*n*+^ trigonal bipyramidal cages (M^III^ = Fe, Cr and Al; M^II^ = Co, Zn and Pd; *n* = 0 for **1–3** and *n* = 6 for **4–5**) of formulae [Fe_2_Co_3_L_6_Cl_6_] (**1**), [Fe_2_Zn_3_L_6_Br_6_] (**2**), [Cr_2_Zn_3_L_6_Br_6_] (**3**), [Cr_2_Pd_3_L_6_(dppp)_3_](OTf)_6_ (**4**) and [Al_2_Pd_3_L_6_(dppp)_3_](OTf)_6_ (**5**) (where HL is 1-(4-pyridyl)butane-1,3-dione and dppp is 1,3-bis(diphenylphosphino)propane) are reported. Neutral cages **1–3** were synthesised using the tritopic [M^III^L_3_] metalloligand in combination with the salts Co^II^Cl_2_ and Zn^II^Br_2_, which both act as tetrahedral linkers. The assembly of the *cis*-protected [Pd^II^(dppp)(OTf)_2_] with [M^III^L_3_] afforded the anionic cages **4–5** of general formula [MIII2PdII3](OTf)_6_. The metallic skeleton of all cages describes a trigonal bipyramid with the M^III^ ions occupying the two axial sites and the M^II^ ions sitting in the three equatorial positions. Direct current (DC) magnetic susceptibility, magnetisation and heat capacity measurements on **1** reveal weak antiferromagnetic exchange between the Fe^III^ and Co^II^ ions. EPR spectroscopy demonstrates that the distortion imposed on the {MO_6_} coordination sphere of [M^III^L_3_] by complexation in the {MIII2MII3} supramolecules results in a small, but measurable, increase of the zero field splitting at M^III^. Complete active space self-consistent field (CASSCF) calculations on the three unique Co^II^ sites of **1** suggest *D*_Co_ ≈ –14 cm^–1^ and *E*/*D* ≈ 0.1, consistent with the magnetothermal and spectroscopic data.

## Abstract 2

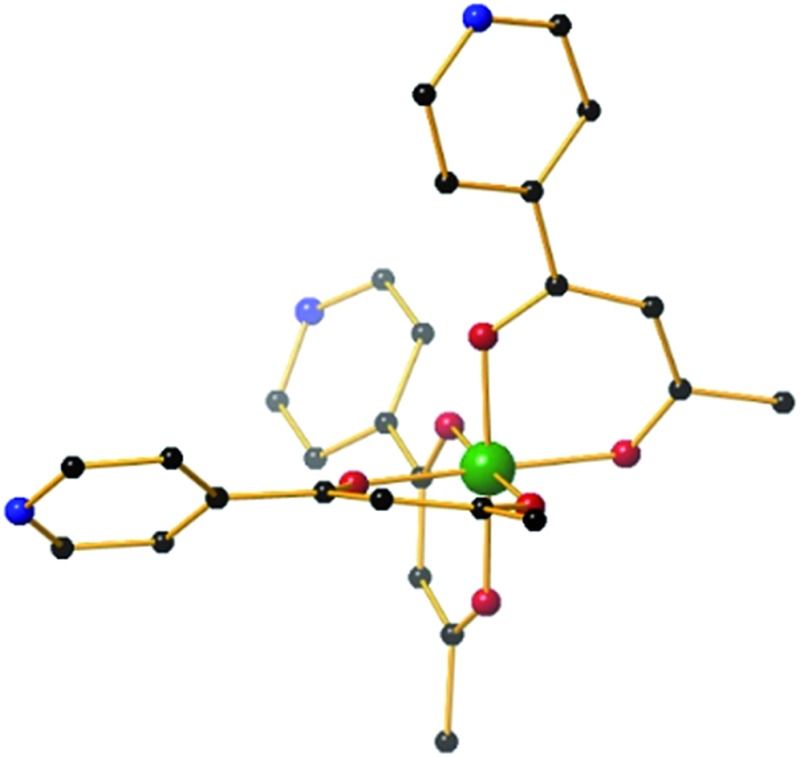
A family of [MIII2MII3]^*n*+^ trigonal bipyramidal cages are characterised in the solution and solid state.

## Introduction

Molecular magnetism relies on the ability of the synthetic chemist to make an enormous breadth of structurally diverse polymetallic cages spanning the d- and f-block of the periodic table.^1–10^ The structural and magnetic characterisation of such species details the magneto-structural relationship and often uncovers fascinating magnetic phenomena which, in turn, feedback into the synthesis of new complexes designed to enhance and improve properties toward application.^11–18^ Synthetic strategies for the design of polymetallic clusters containing multiple paramagnetic metal ions span the range from serendipitous self-assembly in which coordinatively flexible metal ions, that can often exist in multiple oxidation states, are combined with organic ligands capable of bridging in numerous ways to form complexes whose absolute structures are difficult to predict, through to a more ‘supramolecular’ approach whereby metal ions with defined coordination geometries are paired with rigid ligands containing donor atoms with a single, predesigned orientation preference that afford, in most cases, a predicted structure. In the field of molecular magnetism, the latter is perhaps best exemplified by cyanometalate chemistry.^19–23^

A similar synthetic approach is followed in the metallosupramolecular chemistry of diamagnetic cages and capsules where the combination of directional metal–ligand bonding and rigorously rigid ligands creates cages with permanent internal cavities capable of hosting guest molecules, constructed primarily for potential application in, for example, catalysis,^24^ the stabilisation of reactive molecules^25^ and photochemistry.^26^ Due to the difficulties associated with performing solution-based one- and two-dimensional NMR spectroscopy on paramagnetic species, where broad signals and a wide chemical shift range are commonplace,^27^ it is perhaps not surprising that the majority of metallosupramolecular chemistry has focused on the use of diamagnetic metal centres, albeit with some notable exceptions.^28^

**Fig. 1:**
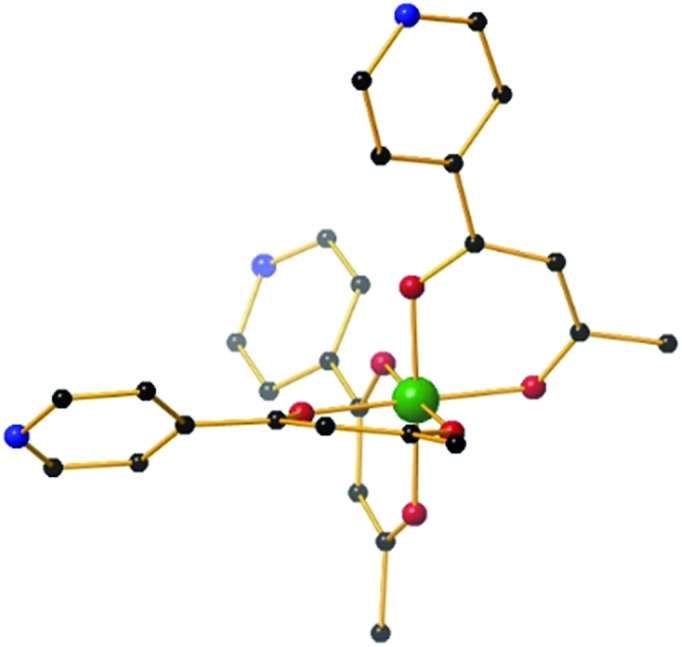
The molecular structure of the *fac*-isomer of the metalloligand [M^III^L_3_] (M = Fe, Cr, Al). Colour code: M^III^ = green, O = red, N = blue, C = black. Hydrogen atoms have been omitted for clarity.

We recently initiated a project that would enable heterometallic, paramagnetic coordination cages to be accessed in a modular and predictable fashion,^29^ an approach centred around the tritopic metalloligand [M^III^L_3_] (where HL = 1-(4-pyridyl)butane-1,3-dione), which features a tris(acac) octahedral transition metal core functionalised with three *p*-pyridyl donor groups (Fig. 1).^30^ Combination of the *fac*-isomer of [M^III^L_3_] with a square-planar M^II^ connector leads to the formation of [MIII8MII6]^*n*+^ molecular cubes.^29,30^ Herein we show that replacement of the square planar connector with tetrahedral or *cis*-capped square planar metal salts leads to the formation of trigonal bipyramidal [MIII2MII3]^*n*+^ cages,^31^ where M^III^ = Fe, Cr, Al and M^II^ = Co, Zn, Pd. Reports of magnetic clusters based on this skeleton are rare, the only previous examples employing cyano bridging ligands.^32–34^

## Experimental section

### Syntheses

1-(4-Pyridyl)butane-1,3-dione (HL) and the metalloligand [Cr^III^L_3_] were prepared according to previously published procedures.^29,35^ All reactions were performed under aerobic conditions. Solvents and reagents were used as received from commercial suppliers. Elemental analyses were carried by Medac Ltd.

### [Fe^III^L_3_]

FeCl_3_ (1 mmol, 0.162 g), 1-(4-pyridyl)butane-1,3-dione (3.5 mmol, 0.57 g) and NaOMe (3.5 mmol, 0.189 g) were dissolved in 100 mL of MeOH/H_2_O (1 : 1 v/v) and left to stir until a red product precipitated (∼24 h). The resultant red precipitate was filtered and washed with water. The crude product was extracted with CHCl_3_ and dried over anhydrous MgSO_4_. The CHCl_3_ was removed under reduced pressure to afford the product as a red solid. Yield (0.46 g, 85%). Elemental analysis (%) calculated (found): C 59.79 (59.53), H 4.46 (4.39), N 7.75 (7.67).

### [Al^III^L_3_]

Al(NO_3_)_3_·9H_2_O (1 mmol, 0.375 g), 1-(4-pyridyl)butane-1,3-dione (3.5 mmol, 0.57 g) and NaOMe (3.5 mmol, 0.189 g) were dissolved in 100 mL of MeOH/H_2_O (1 : 1 v/v) and left to stir until a white product precipitated (∼1 h). The resultant white precipitate was filtered and washed with water. The crude product was extracted with CHCl_3_ and dried over anhydrous MgSO_4_. The CHCl_3_ was removed under reduced pressure to afford the product as a white solid. Yield (0.39 g, 76%). Elemental analysis (%) calculated (found): C 63.16 (63.06), H 4.71 (4.53), N 8.18 (8.11).

### [Fe_2_Co_3_L_6_Cl_6_] (**1**)

To a solution of the metalloligand [Fe^III^L_3_] (108 mg, 0.2 mmol) in 35 mL of acetone, was added CoCl_2_ (39 mg, 0.3 mmol). The resultant mixture was stirred for 30 minutes, before being filtered and layered with Et_2_O. Orange, plate-shaped X-ray quality crystals were obtained after 20 days. Yield (98 mg, 67%). Elemental analysis (%) calculated for C_54_H_48_N_6_O_12_Cl_6_Fe_2_Co_3_: C 44.00, H 3.28, N 5.70. Found: C 44.12, H 3.39, N 5.77.

### [Fe_2_Zn_3_L_6_Br_6_] (**2**)

To a solution of the metalloligand [Fe^III^L_3_] (108 mg, 0.2 mmol) in 35 mL of dichloromethane/acetone (1 : 1 v/v) was added ZnBr_2_ (67 mg, 0.3 mmol). The solution was stirred for 30 minutes, before being evaporated to dryness. The dark-red product was re-dissolved in nitromethane, filtered and allowed to stand. Dark-red, prism-shaped X-ray quality crystals were obtained after room temperature evaporation of the mother liquor after 10 days. Yield (133 mg, 76%). Elemental analysis (%) calculated for C_54_H_48_N_6_O_12_Br_6_Fe_2_Zn_3_: C 36.85, H 2.75, N 4.77. Found: C 36.97, H 2.87, N 4.91.

### [Cr_2_Zn_3_L_6_Br_6_] (**3**)

To a solution of the metalloligand [Cr^III^L_3_] (108 mg, 0.2 mmol) in 35 mL of dichloromethane was added ZnBr_2_ (67 mg, 0.3 mmol). After 1 hour of reaction a precipitate appeared. The dark-red solid product was isolated by filtration, re-dissolved in DMF and layered with MeOH. Dark-red, prism-shaped X-ray quality crystals were obtained after 10 days. Yield (142 mg, 81%). Elemental analysis (%) calculated for C_54_H_48_N_6_O_12_Br_6_Cr_2_Zn_3_: C 37.01, H 2.76, N 4.80. Found: C 36.92, H 2.67, N 4.67.

### [Cr_2_Pd_3_L_6_(dppp)_3_](OTf)_6_ (**4**)

To a solution of the metalloligand [Cr^III^L_3_] (108 mg, 0.2 mmol) in 35 mL of methanol was added [Pd(dppp)_2_(CF_3_SO_3_)_2_] (245 mg, 0.3 mmol; dppp is 1,3-bis(diphenylphosphino)propane). The solution was stirred for 30 minutes, before being filtered and allowed to stand. Orange, rod-shaped X-ray quality crystals were obtained after room temperature evaporation of the mother liquor after 5 days. Yield (275 mg, 78%). Elemental analysis (%) calculated for C_141_H_126_O_30_N_6_F_18_P_6_S_6_Cr_2_Pd_3_: C 48.00, H 3.60, N 2.38. Found: C 47.89, H 3.47, N 2.27.

### [Al_2_Pd_3_L_6_(dppp)_3_](OTf)_6_ (**5**)

To a solution of the metalloligand [Al^III^L_3_] (103 mg, 0.2 mmol) in 35 mL of acetonitrile was added [Pd(dppp)_2_(CF_3_SO_3_)_2_] (245 mg, 0.3 mmol). The solution was stirred for 15 hours at 50 °C, before being filtered and layered with diethyl ether. Colourless, rod-shaped X-ray quality crystals were obtained after 5 days. Yield (288 mg, 83%).^1^H NMR (500 MHz, CD_3_CN): *δ* 8.61 (bs, 12H, Py-*H*), 7.79–7.67 (m, 12H, dppp-Ar*H*), 7.48–7.44 (m, 12H, dppp-Ar*H*), 7.42–7.39 (m, 12H, dppp-Ar*H*), 7.34–7.27 (m, 12H, dppp-Ar*H*), 7.26–7.22 (m, 12H, dppp-Ar*H*), 7.18 (d, *J* = 6.5 Hz, 12H, Py-H), 6.13 (s, 6H, COC*H*CO), 3.28–3.11 (m, 6H, dppp-C*H*_2_), 3.10–2.92 (m, 6H, dppp-C*H*_2_), 2.60–2.36 (m, 3H, dppp-C*H*_2_), 2.15 (s, 18H, C*H*_3_), 1.93–1.75 (m, 3H, dppp-C*H*_2_) ppm. ^13^C NMR (126 MHz, CD_3_CN): *δ* 198.79, 177.61, 151.26, 147.79, 134.91–134.82 (m, 2 signals), 133.70, 133.07–132.99 (m, 3 signals), 130.60–130.51 (m, 2 signals), 130.42–130.33 (m, 2 signals), 127.58–126.88 (m), 125.52–124.82 (m), 124.37, 122.06 (q, *J* = 321.0 Hz), 99.58, 28.10, 22.25–21.92 (m), 18.30 ppm. ^31^P NMR (202 MHz, CD_3_CN) *δ* 6.97 ppm. ^19^F NMR (471 MHz, CD_3_CN) *δ* –79.05 ppm. Diffusion coefficient (DOSY, 500 MHz, CD_3_CN, 298 K) 5.99 × 10^–10^ m^2^ s^–1^, hydrodynamic radius 9.9 Å. ESI TOF HRMS *m*/*z*: found 1010.1238 [M – 3OTf^–^]^3+^, calculated for [C_138_H_126_Al_2_F_9_N_6_O_21_P_6_Pd_3_S_3_]^3+^ 1010.1069. Elemental analysis (%) calculated for C_141_H_126_O_30_N_6_F_18_P_6_S_6_Al_2_Pd_3_: C 48.69, H 3.65, N 2.42. Found: C 48.42, H 3.57, N 2.35.

### Crystal structure information

For compounds **1**, **2** and **3** single-crystal X-ray diffraction data were collected at *T* = 100 K on a Rigaku AFC12 goniometer equipped with an enhanced sensitivity (HG) Saturn 724+ detector mounted at the window of an FR-E+ Superbright MoKα rotating anode generator with HF Varimax optics (70 μm focus)^36^ using Rigaku Crystal Clear and CrysalisPro software^37,38^ for data collection and reduction. The crystals were sensitive to solvent loss and were therefore ‘cold-mounted’ using X-Temp 2 System apparatus at *T* = 70 °C and then quickly transferred to diffractometer.

For compounds **4** and **5** single crystal X-ray diffraction data were measured on a Rigaku Oxford Diffraction SuperNova diffractometer using Cu radiation at *T* = 120 K. The CrysalisPro software package was used for instrument control, unit cell determination and data reduction.^39^ Unit cell parameters in all cases were refined against all data. Crystal structures were solved using the charge flipping method implemented in SUPERFLIP^40^ (**1**, **2**, and **3**), or by direct methods with ShelXS (**4** and **5**). All structures were refined on *F*_o_^2^ by full-matrix least-squares refinements using ShelXL^41^ within the OLEX2 suite.^42^ All non-hydrogen atoms were refined with anisotropic displacement parameters, and all hydrogen atoms were added at calculated positions and refined using a riding model with isotropic displacement parameters based on the equivalent isotropic displacement parameter (*U*_eq._) of the parent atom. All five structures contain accessible voids and channels that are filled with diffuse electron density belonging to uncoordinated solvent, and CF_3_SO_3_^–^ anions in the case of compounds **4–5**. The SQUEEZE routine of PLATON^43^ was used to remove remaining electron density corresponding to solvent and anions not reported in the calculated formula. Crystallographic summary and structure refinement details are presented in Table 1. CCDC: ; 1520425–1520429.†

**Table 1:** X-ray data collection and refinement details

	**1**	**2**·2MeNO_2_	**3**·2MeOH	**4**·17MeOH	**5**·6CH_3_CN
Formula	C_54_H_48_N_6_O_12_Cl_6_Fe_2_Co_3_	C_56_H_48_Br_6_Fe_2_N_8_O_16_Zn_3_	C_56_H_48_Br_6_Cr_2_N_6_O_14_Zn_3_	C_158_H_194_Cr_2_F_18_N_6_O_47_P_6_Pd_3_S_6_	C_153_H_144_N_12_O_30_F_18_Al_2_P_6_S_6_Pd_3_
MWt [g mol^–1^]	1474.17	1876.29	1816.57	4072.56	3724.13
*T* [K]	100	100	100	120	120
*λ* [Å]	0.71075	0.71075	0.71075	1.5418	1.5418
Crystal system	Trigonal	Trigonal	Trigonal	Triclinic	Cubic
Space group	*P*3_2_21	*P*3_2_21	*P*3_2_21	*P*1	*I*43*d*
Unit cell [Å/°]	*a* = 12.7708(5)	*a* = 12.8153(16)	*a* = 13.2429(10)	*a* = 18.4407(9)	*a* = 43.73712(7)
	*b* = 12.7708(5)	*b* = 12.8153(16)	*b* = 13.2429(10)	*b* = 22.0037(9)	*b* = 43.73712(7)
	*c* = 39.0709(12)	*c* = 12.8153(16)	*c* = 38.380(3)	*c* = 27.1925(10)	*c* = 43.73712(7)
	*α* = 90	*α* = 90	*α* = 90	*α* = 104.146(3)	*α* = 90
	*β* = 90	*β* = 90	*β* = 90	*β* = 109.298(4)	*β* = 90
	*γ* = 120	*γ* = 120	*γ* = 120	*γ* = 95.522(4)	*γ* = 90
Volume [Å^3^]	5518.5(5)	5520.9(16)	5829.1(10)	9907.3(8)	83 666.3(4)
*Z*	3	3	3	2	16
Density (calculated) [g cm^–3^]	1.333	1.693	1.546	1.365	1.183
*μ* [mm^–1^]	1.318	4.668	4.322	4.843	3.770
Reflections collected	21 773	32 005	8827	66 752	498 156
Independent reflections	8331	6717	8827	12 021	14 608
*R*_int_	0.1233	0.0627	0.0356	0.0871	0.0934
Goodness-of-fit on *F* ^2^	1.020	1.040	0.971		
Final *R* indices [*F* ^2^ > 2*σ*(*F* ^2^)]	0.0732	0.0379	0.0512	0.0806	0.0869
*R* indices (all data)	0.0887	0.0409	0.0605	0.1050	0.0897

### Physical measurements

Magnetisation measurements were carried out on a Quantum Design SQUID MPMS-XL magnetometer, operating between 1.8 and 300 K for DC applied magnetic fields ranging from 0 to 5 T. Microcrystalline samples were dispersed in eicosane in order to avoid torquing of the crystallites. Heat capacity measurements were carried out for temperatures down to *ca.* 0.3 K by using a Quantum Design 9T-PPMS, equipped with a ^3^He cryostat. The experiments were performed on thin pressed pellets (*ca.* 1 mg) of a polycrystalline sample, thermalised by *ca.* 0.2 mg of Apiezon N grease, whose contribution was subtracted by using a phenomenological expression. X- and Q-band EPR spectra were collected on powdered microcrystalline samples of [FeL_3_] and compounds **1–4** at the UK National EPR Facility in Manchester.

## Results and discussion

### Solution self-assembly and structure

**Fig. 2:**
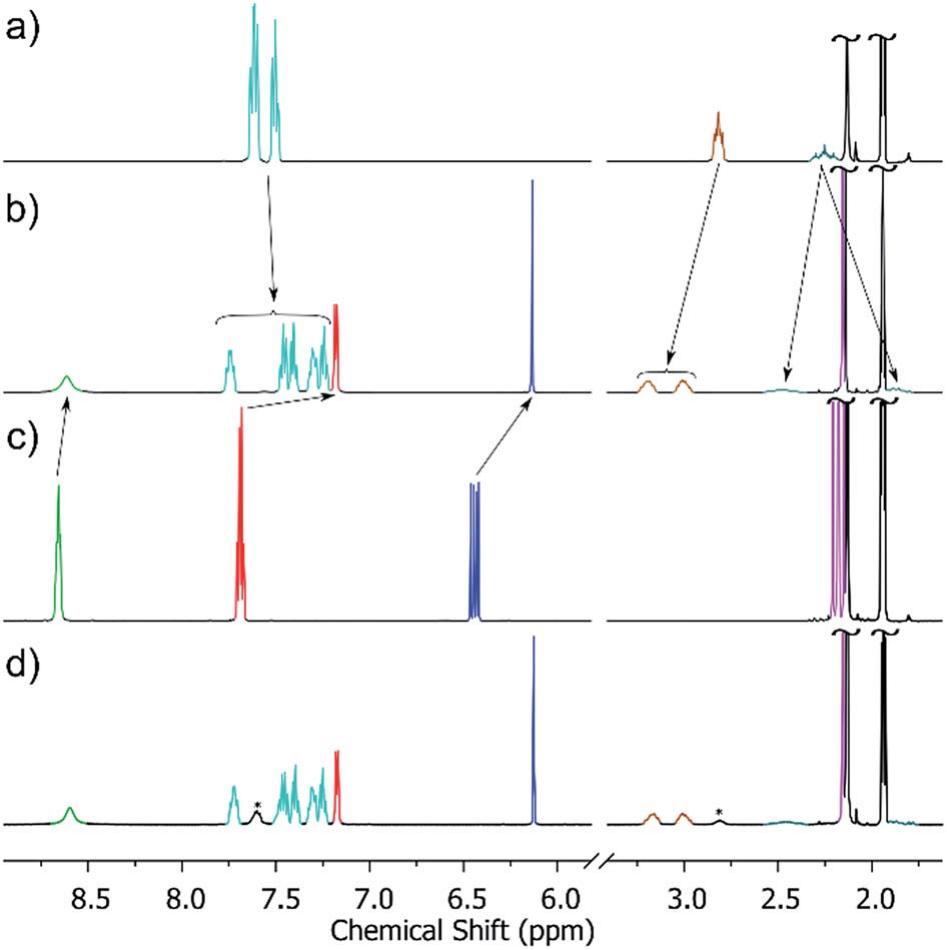
Partial ^1^H NMR spectra (CD_3_CN, 500 MHz, 300 K) of (a) [Pd(dppp)(CF_3_SO_3_)_2_]; (b) cage **5** (re-dissolved crystalline material) (c) [Al^III^L_3_]; (d) the crude self-assembly reaction between a slight excess of [Pd(dppp)_2_(CF_3_SO_3_)_2_] and [Al^III^L_3_] in CD_3_CN (signals for excess [Pd(dppp)_2_(CF_3_SO_3_)_2_] marked *). Colour code: *o*-Py, green; *m*-Py, red; dppp ArH, turquoise; acac CH, blue; dppp-CH_2_, brown and pale blue; acac-CH_3_, magenta.

It could be reasonably expected that reaction of the metalloligand [Al^III^L_3_] with a *cis*-protected square planar complex should yield a trigonal bipyramid. However, in the case of the archetypal 90° acceptor complex [(en)Pd(NO_3_)_2_],^44^ it had previously been shown that instead, displacement of the bidentate ethylene diamine ligand occurs to yield the [Al_8_Pd_6_]^12+^ cube.^29*b*^ We were thus pleased to find that when we switched to the more strongly coordinating bis(diphenylphosphino)propane (dppp), we were able to isolate the [Al_2_Pd_3_]^6+^ trigonal bipyramidal complex, **5**, in 83% yield following reaction overnight at 50 °C between [Al^III^L_3_] and [Pd(dppp)(OTf)_2_] in acetonitrile. All the spectroscopic data indicate that the structure of **5**, confirmed by X-ray crystallography (see below), is preserved in solution. As well as ESI-MS, which reveals the 3+ charge state corresponding to [**5** – 3OTf]^3+^ matching the expected isotopic distribution (see ESI†), the ^1^H NMR spectrum of the product (Fig. 2b) shows just a single set of signals. The ^1^H DOSY spectrum also indicates that all the resonances possess the same diffusion coefficient, which corresponds to a hydrodynamic radius of 9.9 Å, closely matching the data obtained by XRD.

It is also interesting to note that the starting metalloligand [Al^III^L_3_] exists as a mixture of the *mer* and *fac* configurations, clearly evidenced by the multiplet for the acac CH and CH_3_ signals in the ^1^H NMR spectrum (Fig. 2c, resonances shown in blue and magenta), which is replaced by a singlet in the crude reaction mixture (Fig. 2d). This indicates that under the conditions of the reaction, [Al^III^L_3_] is configurationally dynamic, and that the self-assembly process amplifies the proportion of the *fac* configuration through the formation of **5**. While *mer* tris(bidentate) octahedral complexes are also known to generate discrete metallosupramolecular cages,^45^ the divergent disposition of the pendant donor groups create larger closed systems, which with a dynamic system such as this will rapidly rearrange to give the entropically more favourable trigonal bipyramid. A comparison of the ^1^H NMR spectra of the re-dissolved crystalline sample of **5** (Fig. 2b) and the crude reaction solution, obtained by treating a slight excess of [Pd(dppp)(OTf)_2_] with [Al^III^L_3_] in CD_3_CN (Fig. 2d), shows that this amplification is not a solid-state packing effect, rather a solution-based effect. The single set of signals in the ^1^H NMR spectrum of the product (Fig. 2b/d) also indicates that **5** is formed with complete diastereoselectivity.^46^ This represents a second tier of self-sorting, which, unusually, involves Pd-mediated heterochiral recognition of Δ and Λ-[Al^III^L_3_] enantiomers (see below).

### Solid-state structure descriptions

**Fig. 3:**
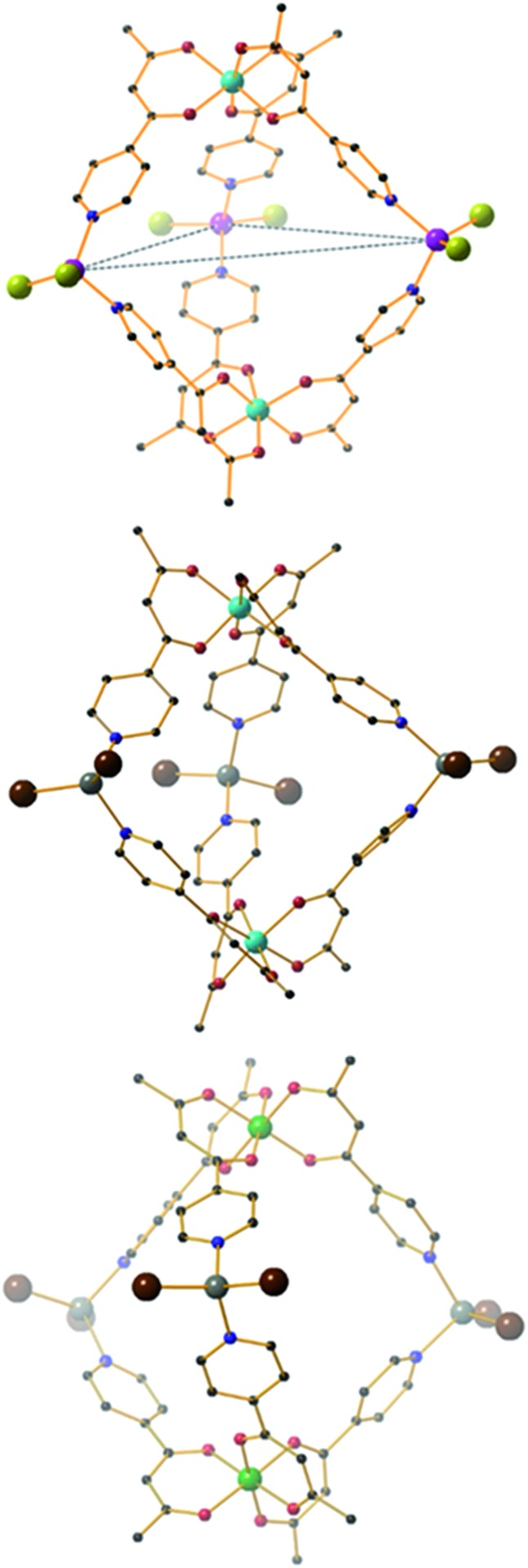
From top to bottom, molecular structures of (ΛΛ)-**1**, **2** and **3**. Colour code: Fe = cyan, Co = magenta, Cr = green, Zn = grey, O = red, N = blue, Cl = green, Br = brown, C = black. H-atoms omitted for clarity. The dashed blue line in the upper figure highlights the trigonal plane of M^II^ ions.

**Fig. 4:**
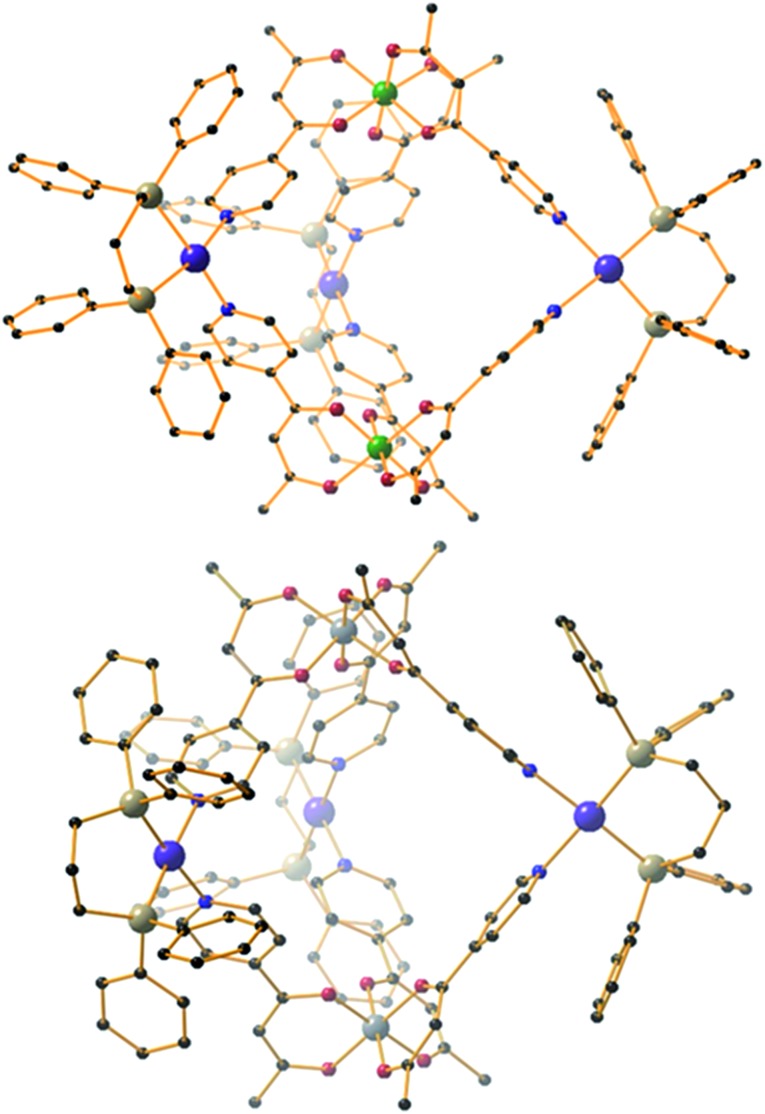
Molecular structures of (ΛΔ)-**4** (top) and **5** (bottom). Colour code: Cr = green, Al = grey, Pd = magenta, P = silver, O = red, N = blue, C = black. H-atoms and OTf^–^ counteranions omitted for clarity.

The heterometallic trigonal bipyramid cages [Fe_2_Co_3_L_6_Cl_6_] (**1**), [Fe_2_Zn_3_L_6_Br_6_] (**2**), [Cr_2_Zn_3_L_6_Br_6_] (**3**), [Cr_2_Pd_3_L_6_(dppp)_3_](OTf)_6_ (**4**) and [Al_2_Pd_3_L_6_(dppp)_3_](OTf)_6_ (**5**) (Fig. 3 and 4) were all synthesised in a similar manner, by addition of either tetrahedral or *cis*-protected square planar M^II^ compounds to the metalloligand [M^III^L_3_] (M^III^ = Fe, Cr or Al) in acetone, methanol, acetonitrile or a mixed solvent system, with crystals isolated from slow evaporation of the mother liquor, or diffusion of Et_2_O or MeOH (see the Experimental section for full details). The metallic skeletons of the cages in **1–5** describe a trigonal bipyramid with the M^III^ ions situated on the axial positions and the M^II^ ions on the equatorial sites. The approximate dimensions of the [MIII2MII3]^*n*+^ metallic skeleton are M^III^···M^II^ (8.77–8.99 Å), M^II^···M^II^ (11.72–12.80 Å) and M^III^···M^III^ (10.75–11.20 Å).

Each of the three M^II^ metal ions is coordinated by two N donors from the pyridyl groups of [M^III^L_3_]. The N–M^II^–N angle of the tetrahedral CoN_2_Cl_2_/ZnN_2_Br_2_ moiety for compounds **1–3** lies in the range 90.63–103.57°; in **4–5** the equivalent N–Pd–N angle is in the range 84.40–85.39°. Each [M^III^L_3_] corner unit consists of a six-coordinate M^III^ ion with regular {MO_6_} octahedral geometry. For the three different [M^III^L_3_] metalloligands used in the synthesis the M^III^–O distances and angles are: Fe–O 1.98–2.02 Å, Fe–O *cis*/*trans* angles 83.48–95.17° and 169.99–178.40°, respectively; Cr–O 1.91–1.98 Å, Cr–O *cis*/*trans* angles 86.82–94.04° and 176.35–179.85°, respectively; Al–O 1.86–1.89 Å, Al–O *cis*/*trans* angles 88.84–91.43° and 179.03–179.54°, respectively. The Co^II^ and Zn^II^ ions lie in distorted tetrahedral environments with bond distances in the range 2.05–2.35 Å (Co–Cl ∼ 2.23 Å, Co–N ∼ 2.05 Å, Zn–Br ∼ 2.35 Å and Zn–N ∼ 2.06 Å) and bond angles around the metal centres ranging from 90.62° to 120.08°. In compounds **4** and **5**, the Pd^II^ ion is 90° *cis*-blocked through the use of the dppp ligand (Pd–P bond distance ∼ 2.27 Å). The coordination of Pd to [M^III^L_3_] through the use of Pd–N bonds (ranging from 2.08–2.14 Å) creates a distorted square planar geometry around the Pd centre with *cis*/*trans* bond angles in the range 84.40–93.50° and 165.49–178.57°, respectively. While complexes **1–3** are neutral, charge balance is maintained in **4** and **5** through the presence of a total of six CF_3_SO_3_^–^ anions, lying outside the cage.

While the intrametallic distances of the five trigonal bipyramids are similar, there is nonetheless a distinct diastereomeric difference between structures **1–3** and **4–5**. Whereas **1–3** are all homochiral racemates in which each intact capsule features two [M^III^L_3_] units that possess the same Λ or Δ chirality, in contrast structures **4** and **5** are both the achiral heterodiastereomer. While sorting of chiral octahedral metal motifs has been frequently observed in metallosupramolecular assembly reactions, for the vast majority homochiral assemblies are energetically preferred.^47^ The commonality of the [Pd(dppp)] unit in both **4** and **5** that feature different [M^III^L_3_] metalloligands would suggest that either the small change in angle between pyridine donors at each M^II^ connector and/or the interactions of the dppp protecting ligand with these donors cause the change in diastereomeric preference. Solution studies with **5** would also indicate this is not simply due to selective crystallization from a complex mixture (see above). Outwith cyanometalate chemistry,^32–34^ compounds **1–4** represent the first examples of trigonal bipyramids built with paramagnetic metal ions, and join a small family of analogous compounds containing diamagnetic metal ions.^48–52^

### SQUID magnetometry

**Fig. 5:**
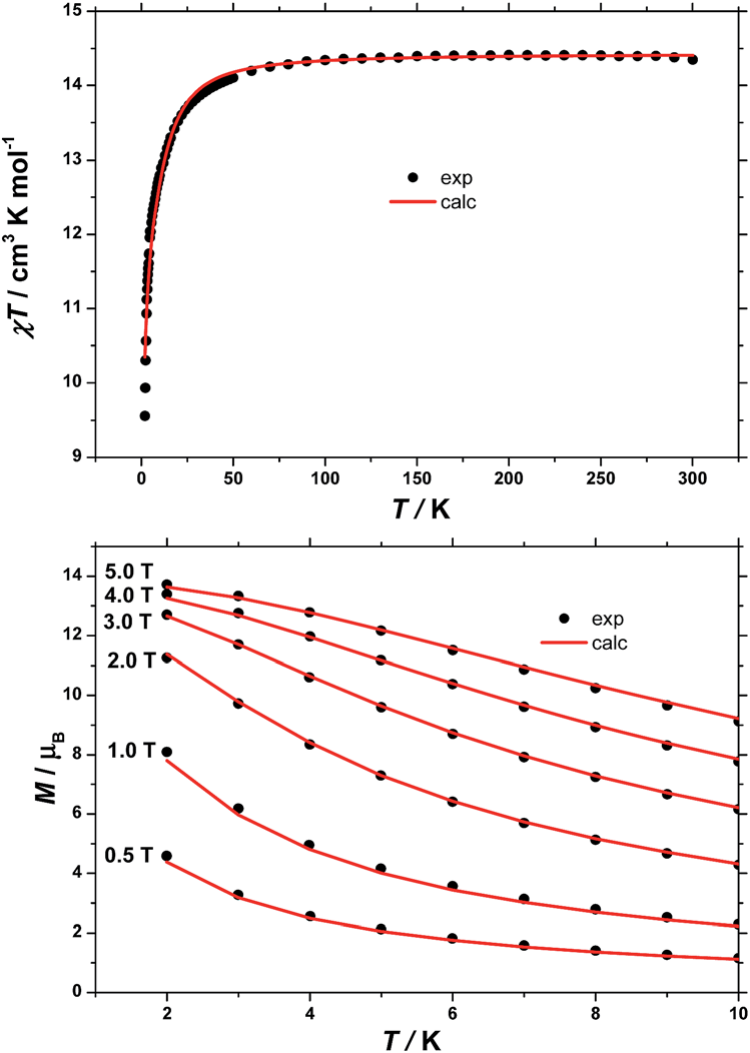
(Top) Temperature dependence of the *χT* product of a polycrystalline sample of **1** with *B* = 0.1 T. (Bottom) VTVB magnetisation data of **1** in the field and temperature ranges 0.5 to 5 T and 2 to 10 K, respectively. Solid lines are the best-fit curves, see text for details.

The dc (direct current) molar magnetic susceptibility, *χ*, of a polycrystalline sample of **1** was measured in an applied magnetic field, *B*, of 0.1 T, over the 2–300 K temperature, *T*, range. The experimental results are shown in Fig. 5 in the form of the *χT* product, where *χ* = *M*/*B*, and *M* is the magnetisation of the sample. At room temperature, the *χT* product of **1** has a value of 14.4 cm^3^ K mol^–1^, in good agreement with the sum of Curie constants for a [FeIII2CoII3] unit (14.375 cm^3^ K mol^–1^, *g*_Fe_ = *g*_Co_ = 2.0). Note that the estimation of the *g*-value of the Co^II^ ions here is an approximation and subject to error (*e.g.* lattice solvent lost upon sample drying will result in a variation of the samples diamagnetism), and a better measure comes from the EPR spectroscopy, which is consistent with *g*_Co_ = 2.3 (*vide infra*). Upon cooling, the *χT* product of **1** remains essentially constant down to approximately 100 K, wherefrom it decreases upon further cooling to 9.5 cm^3^ K mol^–1^ at 2 K. Given that the anisotropy of Fe^III^ is negligible, this behaviour is consistent with a relatively large single-ion magnetic anisotropy for the Co^II^ centres and/or an antiferromagnetic exchange interaction between the Fe^III^ and Co^II^ centres. To better define the low-temperature magnetic properties of **1**, low temperature variable-temperature-and-variable-field (VTVB) magnetisation data were measured in the temperature and magnetic field ranges *T* = 2–12 K and *B* = 0–5 T (Fig. 5). At the highest investigated field (5 T) and the lowest investigated temperature (2 K), the magnetisation of **1** is of 13.7 *μ*_B_ (*μ*_B_ is the Bohr magneton). Furthermore, when the VTVB data of **1** are plotted against the reduced quantity *μ*_B_ *B*/*kT*, little nesting of the VTVB data is observed. This observation indicates that the part of the energy spectrum of **1** probed under these experimental conditions does not present significant anisotropy splitting with respect to the temperature of measurement at zero magnetic field.

For the quantitative interpretation of the magnetisation data, we used spin-Hamiltonian (1)1
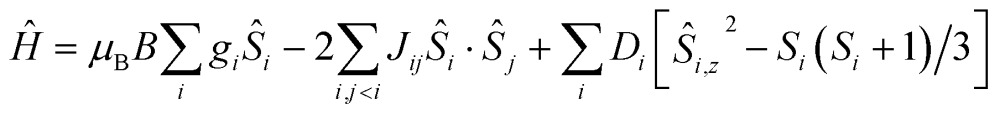
where the summation indexes *i*, *j* run through the constitutive metal centres, *g*_i_ is the *g*-factor of the *i* ^th^ centre, *ŝ* is a spin operator, *J* is the isotropic exchange interaction parameter, *D* is the uniaxial anisotropy parameter and *S* is the total spin.

**Fig. 6:**
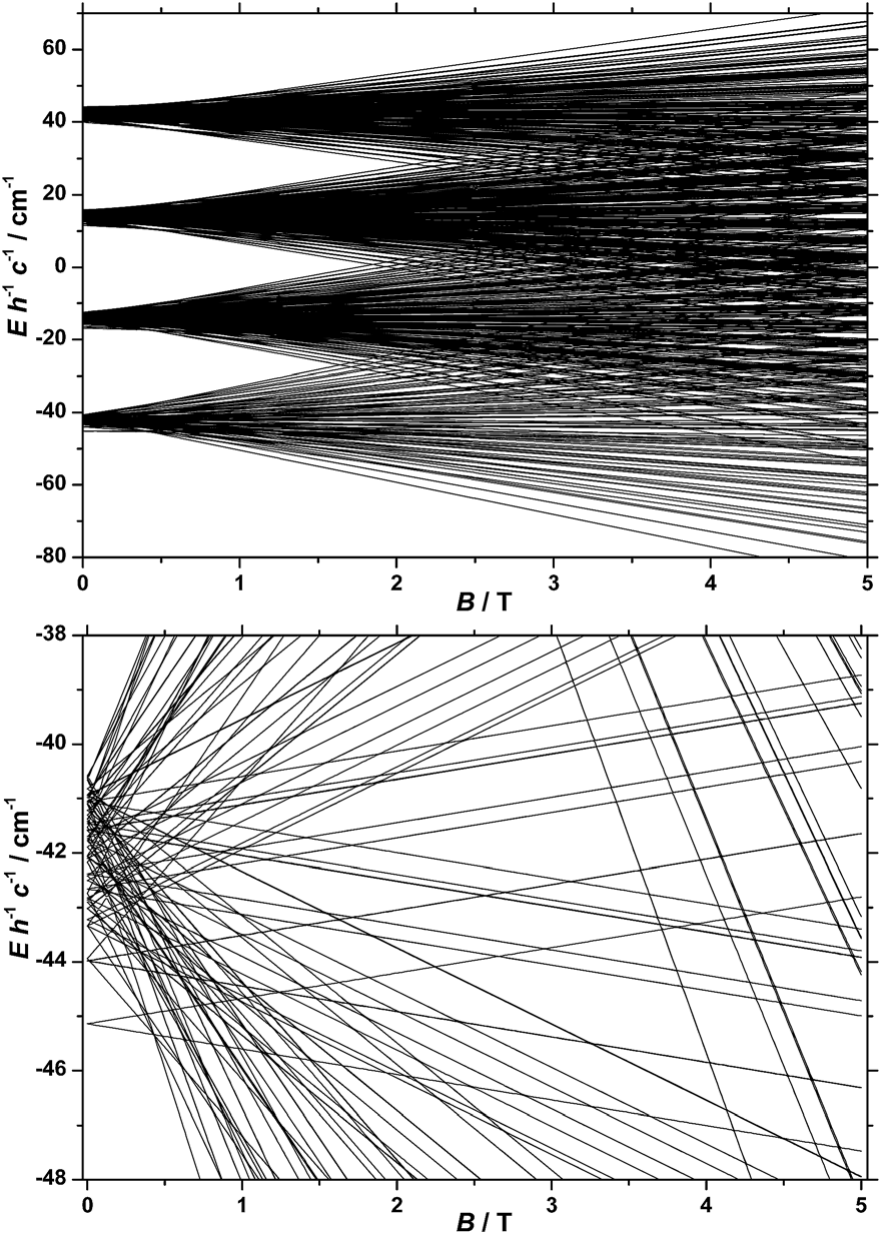
(Top) Energy spectrum of **1** determined with the best-fit parameters (see text) and the magnetic field applied along the quantisation axis. (Bottom) Low-lying states of the energy spectrum of **1**, determined as described in the text.

In our spin-Hamiltonian model, we assume for simplicity that all *g*-factors are equal to 2, *S*_Fe^III^_ = 5/2, *S*_Co^II^_ = 3/2, we only consider exchange interactions between Co^II^ and Fe^III^ centres, and neglect the single-ion anisotropy of Fe^III^. Furthermore, we fix the uniaxial anisotropy of Co^II^ to *D*_Co_ = –14 cm^–1^, as extracted from the modelling of the EPR data and theoretical calculations, which are discussed further in the following sections. Thus, at this point our model contains only one free parameter, namely, the isotropic exchange between Fe^III^ and Co^II^, *J*_Fe–Co_. The *χT* product of **1** was fitted to spin-Hamiltonian (1) by full matrix numerical diagonalisation of the spin-Hamiltonian of the full system of dimension 2304 by 2304, through use of the Levenberg–Marquardt algorithm.^53^ This resulted in the best-fit parameter *J*_Fe–Co_ = –0.04 cm^–1^. In order to verify the validity of our model, *J*_Fe–Co_ was fixed to the determined best-fit value, *J*_Fe–Co_ = –0.04 cm^–1^, and *D*_Co_ was maintained fixed at –14 cm^–1^. At this point our model contains no free parameters. Thereafter, the VTVB data of **1** were simulated by use of spin-Hamiltonian (1). The simulated curves are shown as solid red lines in Fig. 5. With these parameters, the energy spectrum of **1** consists of four groups of densely packed states, each separated by approximately 2*D*_Co_ (Fig. 6). It is interesting to note that multiple ground level crossings simultaneously occur at approximately 0.47 T when the magnetic field is applied parallel to the quantisation axis.

### Heat capacity

**Fig. 7:**
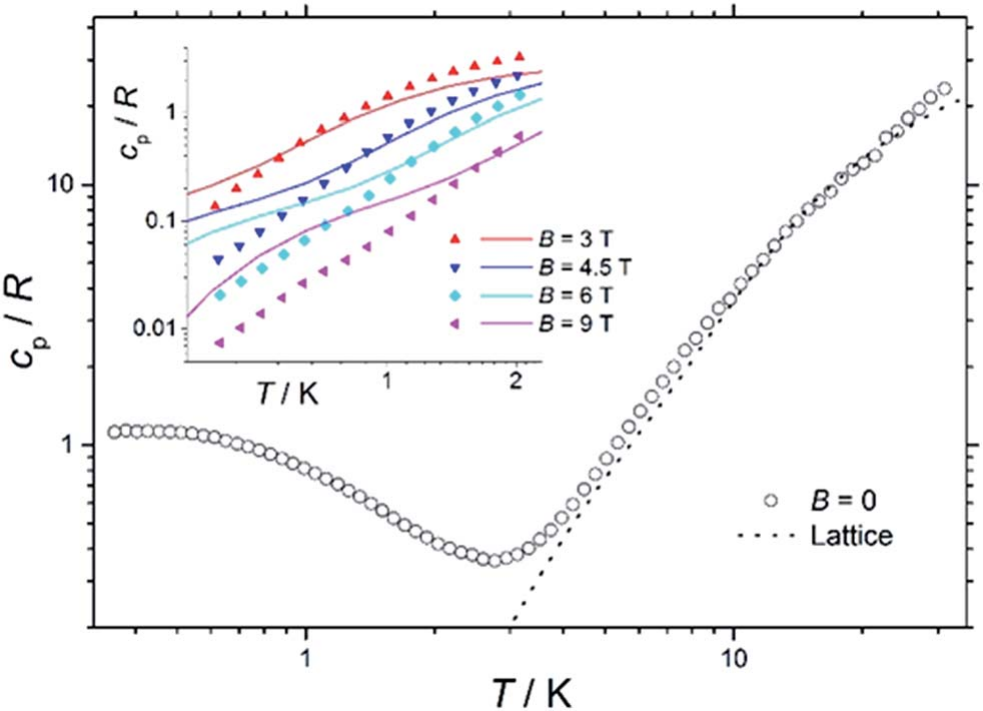
Temperature dependence of the zero-field heat capacity *c*_p_, normalised to the gas constant *R*, for a polycrystalline sample of **1**. The dotted line is the lattice contribution. Inset: temperature dependence of *c*_p_/*R* of **1** for *T* < 2 K and *B* ≥ 3 T. Solid lines are the best-fit curves, see text for details.

Fig. 7 shows the collected heat capacity data, normalised to the gas constant, *c*_p_/*R* of **1** as a function of temperature (between *ca.* 0.3 K and 30 K) for zero-applied magnetic field. As is typical for molecular magnetic materials,^54^ lattice vibrations contribute predominantly to *c*_p_ as a rapid increase above liquid-helium temperature. The lattice contribution can be described by the Debye model (dotted line in Fig. 7), which simplifies to a *c*_p_/*R* = *aT* ^3^ dependence at the lowest temperatures, where *a* = 7.6 × 10^–3^ K^–3^ for **1**.

For *T* < *ca.* 3 K, the zero-field *c*_p_ shows a wide bump-like anomaly, which we attribute to the splitting of the spin levels by zero-field splitting and magnetic interactions. At such low temperatures, the magnetic measurements are very sensitive to the applied magnetic field, as seen in the experimental behaviour for fields of 3 T and higher (inset of Fig. 7). Such large intensities of the applied magnetic field are sufficient for promoting full decoupling between the individual spin centres (we recall that the exchange interaction is as small as *J*_Fe–Co_ = –0.04 cm^–1^ on the basis of the fit of the magnetometry data). Therefore, the temperature and field dependence of the *c*_p_ data in Fig. 7 (inset), collected for *B* ≥ 3 T, are particularly suitable for probing the influence of crystal fields on **1**, down to temperatures significantly lower than the ones obtained in the magnetisation measurements.

The solid lines in Fig. 7 are the curves calculated for Hamiltonian (1), using the best-fit parameters from the magnetothermal and spectroscopic data and theoretical calculations, *i.e.*, *D*_Co_ = –14 cm^–1^ and the here-negligible *J*_Fe–Co_ = –0.04 cm^–1^. The agreement with the experimental data is good, though not outstanding. Anticipating the discussion on the EPR spectra (*vide infra*), we have checked that adding a zero-field splitting (ZFS) of *D*_Fe_ = –0.2 cm^–1^ at the Fe^III^ sites does not improve the fit. The discrepancy is most evident below *ca.* 1 K, where the experimental data have lower values than the calculated ones. This behaviour can be explained by a wider broadening of the low-lying energy spectrum, likely induced by higher-order anisotropy terms, which are not taken into account in Hamiltonian (1).

### EPR spectroscopy

We previously reported EPR spectra of [CrL_3_], giving the ZFS of the Cr^III^, *s* = 3/2 ion as *D* = –0.55 cm^–1^ with a small rhombicity of |*E*/*D*| = 0.045.^29^ Q-Band spectra of **3** and **4** are similar to that of [Cr^III^L_3_], and give *D* = –0.64 and –0.61 cm^–1^, respectively (Fig. S11;† |*E*/*D*| = 0.03–0.04).^55^ Hence, the distortion imposed on the {CrO_6_} coordination sphere of [Cr^III^L_3_] by complexation in the {CrIII2MII3} supramolecules results in a small, but measurable, increase of the ZFS at Cr^III^. The {CrO_6_} metric parameters do not appear to be very different.

**Fig. 8:**
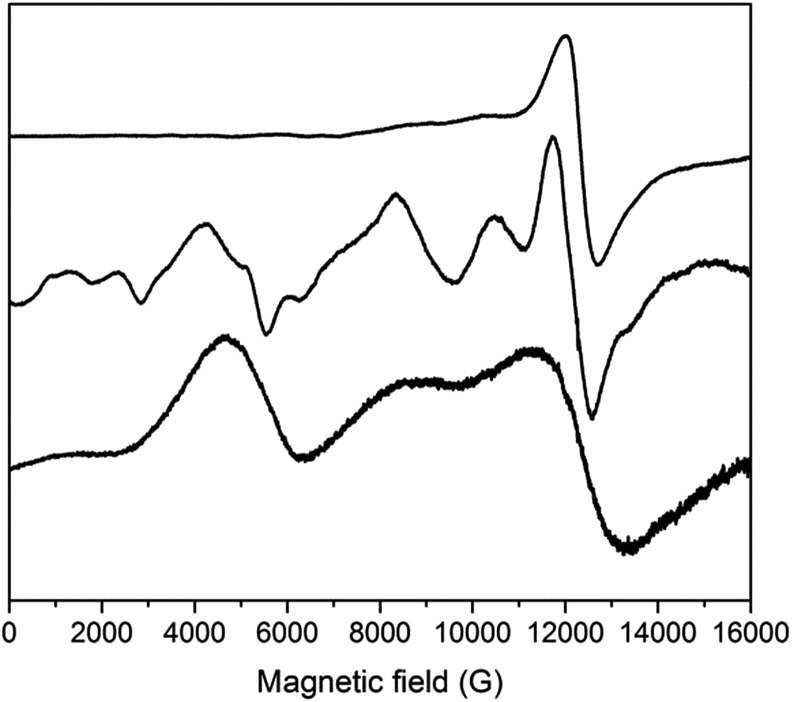
Q-Band EPR spectra of powdered samples of (from top to bottom) [FeL_3_], **2** and **1** at 5 K.

Such an increase in *D* is also found for the Fe^III^ (*s* = 5/2) systems. X- and Q-band EPR spectra of [Fe^III^L_3_] reveal a rather small ZFS of *D* = 0.08 cm^–1^ with |*E*/*D*| = 1/3 (Fig. 8 and S12;† note the sign of *D* has no significance with a fully rhombic *D*-tensor). These values are similar to those reported for [Fe(acac)_3_] (|*D*| = 0.16 cm^–1^, *E*/*D* = 0.3)^56^ and [Fe(dpm)_3_] (dpm = dipivaloylmethane; *D* = –0.20 cm^–1^, |*E*/*D*| = 0.25).^57^ On incorporation into the {FeIII2ZnII3} complex **2**, a much richer spectrum is observed (Fig. 8 and S12), giving *D* = 0.20 cm^–1^ (*E*/*D* = 1/3). Angular overlap model studies on [Fe(acac)_3_] and [Fe(dpm)_3_] show *D* to be very sensitive to the trigonal distortion at Fe^III^,^57^ and there is a more significant structural difference in the {Fe^III^O_6_} coordination spheres when bound in {FeIII2ZnII3}, with longer Fe–O bonds and wider O–Fe–O angles in the {py}_3_ face, than in the equivalent Cr^III^ systems.

The {FeIII2CoII3} complex **1** gives Q-band EPR spectra with very broad features at *ca.* 5, 9 and 12 kG that line up with the main features of the spectrum of the {FeIII2ZnII3} complex **2**. Hence, the ZFS at Fe^III^ must be similar. The very large ZFS at Co^II^ means that only transitions within the ground Kramers doublet of this ion are observed (the microwave energy, *hν* ≪ |*D*|), and there must be a significant rhombicity in order for these transitions to fall within the observed features. The spectra also show that the *J*_FeCo_ exchange interaction must be very weak, resulting only in severe broadening of the peaks. Test calculations on a simple {Fe^III^Co^II^} model, with fixed ZFS at the *s* = 5/2 and 3/2 spins (the latter taking *D* = –14 cm^–1^ with *E*/*D* = 0.1; averaging the results of CASSCF calculations – see below) suggest that if |*J*_FeCo_| > *ca.* 0.02 cm^–1^ then additional features would be observed in the Q-band EPR spectrum. Note that the limit for the full, five-spin system would be different.

The *D*_Fe_ values obtained from EPR would have a negligible effect on the calculated *χT*(*T*) and *c*_p_(*T*,*B*) curves for **1**, and a negligible effect on the global level structure in Fig. 6a, because both |*D*_Fe_| and |*J*_FeCo_| are ≪|*D*_Co_|. However, it would affect the detail of the states within each of the densely packed multiplets of Fig. 6a, because |*D*_Fe_| and |*J*_FeCo_| are of similar magnitude.

### Theoretical studies

**Fig. 9:**
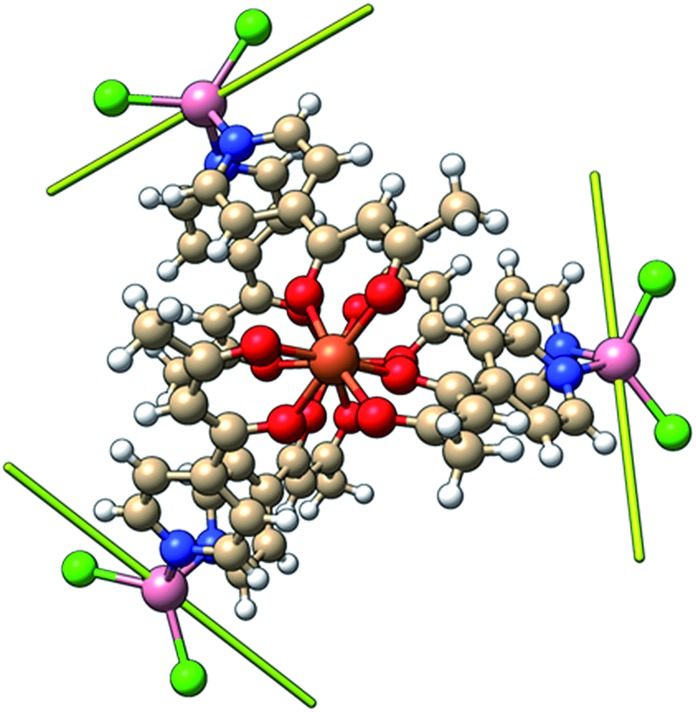
Orientation of the principal anisotropy axis for the Co^II^ sites in **1** (yellow rods); orange = Fe, pink = Co, green = Cl, red = O, blue = N, beige = C, white = H.

In order to independently verify the large ZFS of Co^II^ we have performed complete active space self-consistent field (CASSCF) calculations on the three unique Co^II^ sites of **1**, see the SI for details. The results suggest *D*_Co_ = –14 cm^–1^, *E*/*D* = 0.1 (Table S1†) which is entirely consistent with the magnetometry and heat capacity data. The calculations also suggest that the principal axes of the local ZFS tensors are oriented roughly perpendicular to the Fe^III^–Fe^III^ axis and canted approximately 120° with respect to one another in the plane (Fig. 9). Accounting for the non-collinearity in spin-Hamiltonian (1) did not improve the quality of the fits to the magnetometry or heat capacity data.

## Conclusions

Complexes **1–5** represent a novel, and unusual family of trigonal bipyramidal cage complexes, built with the tritopic [ML_3_] metalloligand, featuring a tris(acac) octahedral transition metal core functionalised with three *p*-pyridyl donor groups, and a series of transition metal salts. Outwith cyanometalate chemistry, compound **1** represents the first example of such a cage containing paramagnetic metal ions. Complementary studies investigating the diamagnetic variants using ^1^H NMR spectroscopy reveal some interesting features about the solution self-assembly process. Firstly, the [M^III^L_3_] metalloligand is a highly dynamic tritopic building block as evidenced by *fac* configurational isomer being amplified at the expense of the *mer* during the course of cage formation. The self-assembly process also occurs with high and unusual stereoselectivity wherein the trigonal bipyramids are formed exclusively from twisted pyramidal components of opposite Δ/Λ-handedness. Solution stability of the cage is also confirmed *via* mass spectrometry. SQUID magnetometry and heat capacity measurements on **1** reveal weak antiferromagnetic exchange between the Fe^III^ and Co^II^ ions, with |*D*_Co_| = 14 cm^–1^. EPR spectroscopy reveals that the distortion imposed on the {MO_6_} coordination sphere of [M^III^L_3_] by complexation in the {MIII2MII3} supramolecules results in a small, but measurable, increase of the zero field splitting at M^III^. CASSCF calculations on the three unique Co^II^ sites of **1** suggest that the principal axes of the local ZFS tensors are oriented perpendicular to the Fe^III^–Fe^III^ axis, but canted ∼120° with respect to each other.
