# Supplementary material for: [MIII2MII3]n+ trigonal bipyramidal cages based on diamagnetic and paramagnetic metalloligands
Source: Chem Sci. 2017 May 19;8(8):5526–35. doi: 10.1039/c7sc00487g (PMC5618769; doi:10.1039/c7sc00487g)
Supplement: Supplementary file 1 [file SC-008-C7SC00487G-s001.pdf]

This journal is © The Royal Society of Chemistry 2017

Instrumentation

Electrospray Ionization (E.S.I.) mass spectra were measured on a Bruker MicroToF 2 for **FeL_3**, **CrL_3** and **AlL_3**, and on a Waters Synapt G2 Q-TOF for compounds **4** and **5**. NMR spectra were recorded on a 500MHz Bruker Avance NMR spectrometer. Inductively Coupled Plasma Optical Emission Spectroscopy (ICP-OES) analysis of compound **1** was performed on a Perkin Elmer Optima 5300 DV ICP-OES employing an RF forward power of 1400 W, with argon gas flows of 15, 0.2 and 0.75 L min^{-1} for plasma, auxiliary, and nebuliser flows, respectively. Powder X-ray diffraction in capillary (0.7 mm) mode were carried out on a Bruker D8 Advance between 5-40 (2θ) at 0.0395 increments. IR (ATR) spectra were performed on a PerkinElmer Spectrum 65 between 4000-400 cm^{-1}.

Infrared Spectroscopy (ATR, cm^{-1})

FeL_3: 3200.86 (broad), 1577.42, 1541.94, 1509.37, 1488.35, 1411.82, 1358.27, 1319.57, 1291.00, 1217.40, 1117.25, 1064.99, 1004.50, 954.33, 843.74, 789.15, 688.38, 605.55, 572.30.

AlL_3: 3296.43 (broad), 1592.43, 1548.91, 1516.40, 1416.83, 1385.90, 1304.75, 1212.30, 1121.39, 1065.30, 1006.12, 963.63, 843.00, 785.34, 699.61, 598.84, 565.13.

(**1**): 3370.23 (broad), 2955.45, 1682.76, 1565.34, 1528.09, 1496.21, 1475.39, 1405.54, 1346.47, 1305.37, 1273.77, 1199.20, 1101.26, 1048.58, 1001.10, 940.66, 834.79, 764.33, 681.68, 564.27.

(**2**): 2967.51, 1576.06, 1542.49, 1507.15, 1489.59, 1425.01, 1374.84, 1358.37, 1316.76, 1289.05, 1212.13, 1115.14, 1062.89, 1026.24, 1000.82, 952.57, 848.68, 806.12, 785.50, 709.82, 696.00, 652.74, 581.29, 558.33.

(**3**): 2972.70, 1694.67, 1564.86, 1530.20, 1497.94, 1477.71, 1413.71, 1371.81, 1346.63, 1303.00, 1279.67, 1199.40, 1048.51, 1013.38, 940.89, 1105.60, 1048.94, 1013.72, 940.38, 835.64, 768.63, 699.59, 685.70, 602.40, 560.46.

(**4**): 3489.97 (broad), 2970.30, 1583.90, 1548.34, 1517.13, 1496.42, 1436.84, 1389.25, 1249.45, 1223.57, 1156.52, 1028.22, 746.41, 692.87, 636.24, 573.77.

(**5**): 3491.76 (broad), 2970.29, 1597.34, 1522.43, 1497.38, 1436.42, 1399.71, 1248.12, 1223.84, 1155.74, 1027.45, 746.57, 692.64, 636.20, 572.26.

Analytical characterisation data for [$Al_2Pd_3L_6(dppp)_3](OTf)_6$ (5)

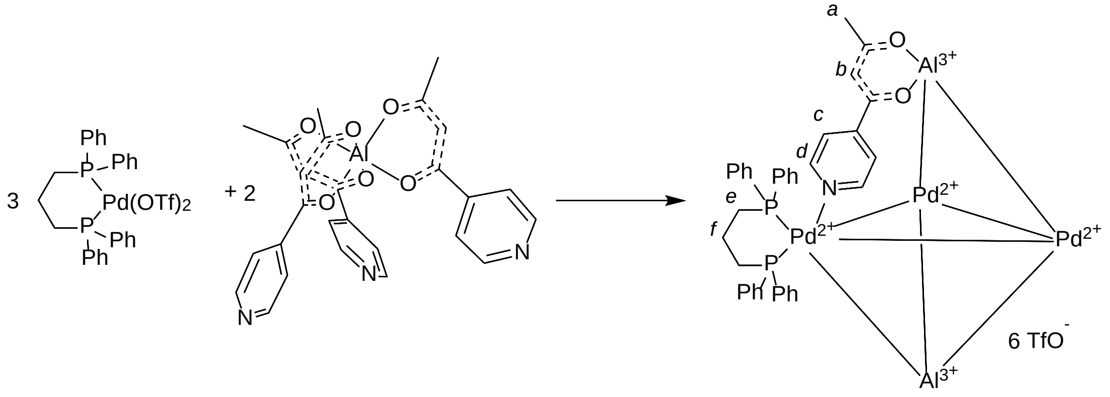

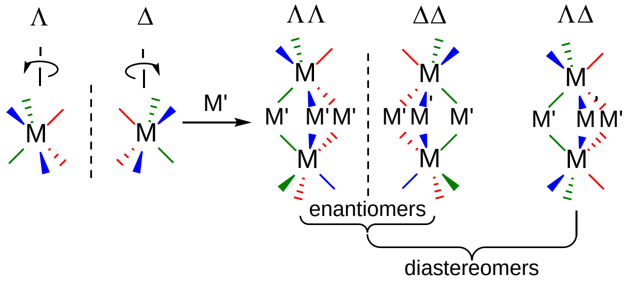

^{1}H NMR (500 MHz, CD_3CN): δ 8.61 (bs, 12H, H_d), 7.79 – 7.67 (m, 12H, Ph), 7.48 – 7.44 (m, 12H, Ph), 7.42 – 7.39 (m, 12H, Ph), 7.34 – 7.27 (m, 12H, Ph), 7.26 – 7.22 (m, 12H, Ph), 7.18 (d, J = 6.5 Hz, 12H, H_c), 6.13 (s, 6H, H_b), 3.28 – 3.11 (m, 6H, H_e), 3.10 – 2.92 (m, 6H, H_e), 2.60 – 2.36 (m, 3H, H_f), 2.15 (s, 18H, H_a), 1.93 – 1.75 (m, 3H, H_f) pp

^{13}C NMR (126 MHz, CD_3CN): δ 198.79, 177.61, 151.26, 147.79, 134.86 (m, 2 signals), 133.70, 133.07 (m, 3 signals), 130.56 (m, 2 signals), 130.38 (m, 2 signals), 127.16 (m), 125.10 (m), 124.37, 122.06 (q, J = 321.0 Hz), 99.58, 28.10, 22.08 (m), 18.30 ppm.

^{31}P NMR (202 MHz, CD_3CN) δ 6.97 ppm. ^{19}F NMR (471 MHz, CD_3CN) δ –79.05 ppm.

Diffusion coefficient (DOSY, 500 MHz, CD_3CN, 298 K) 5.99 × 10^{-10} m^2/s, Hydrodynamic radius 9.9 Å.

ESI TOF HRMS m/z: Found 1010.1238 [M – 3 $OTf^-]^{3+}$, calculated for $[C_{138}H_{126}Al_2F_9N_6O_{21}P_6Pd_3S_3]^{3+}$ 1010.1069.

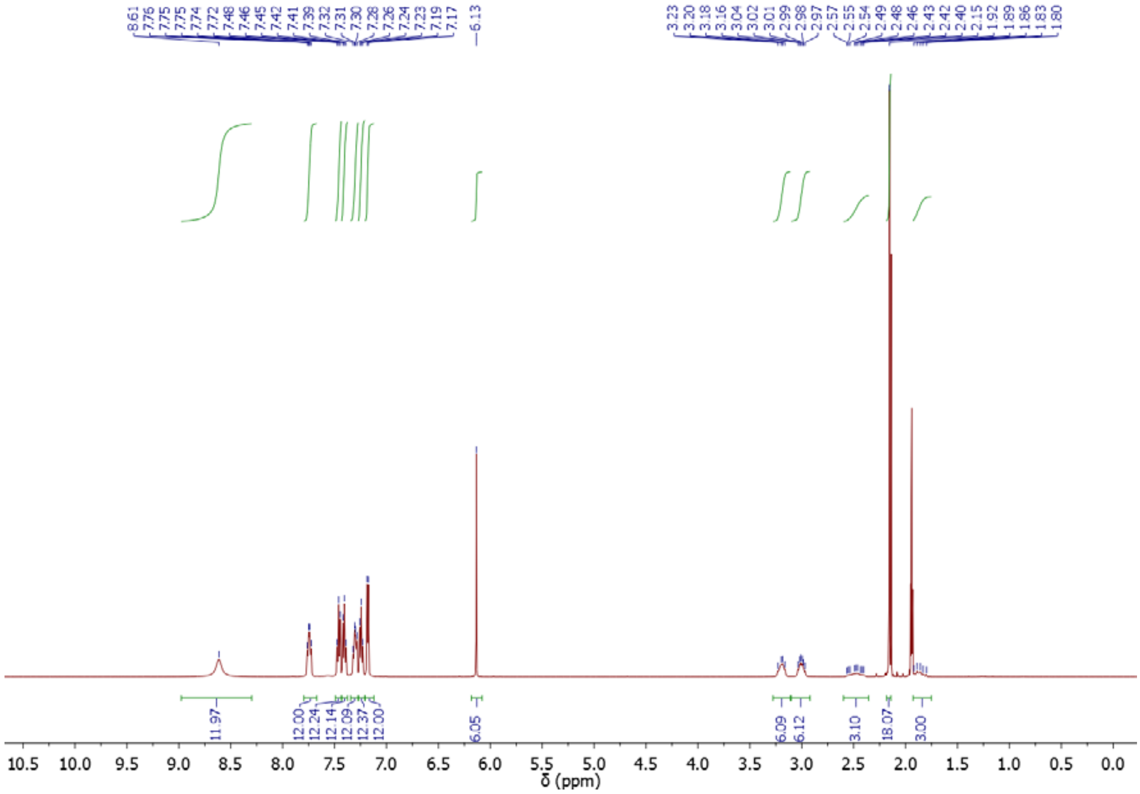

Figure S1. ^{1}H NMR (500 MHz, CD_3CN, 300 K) of **5**.

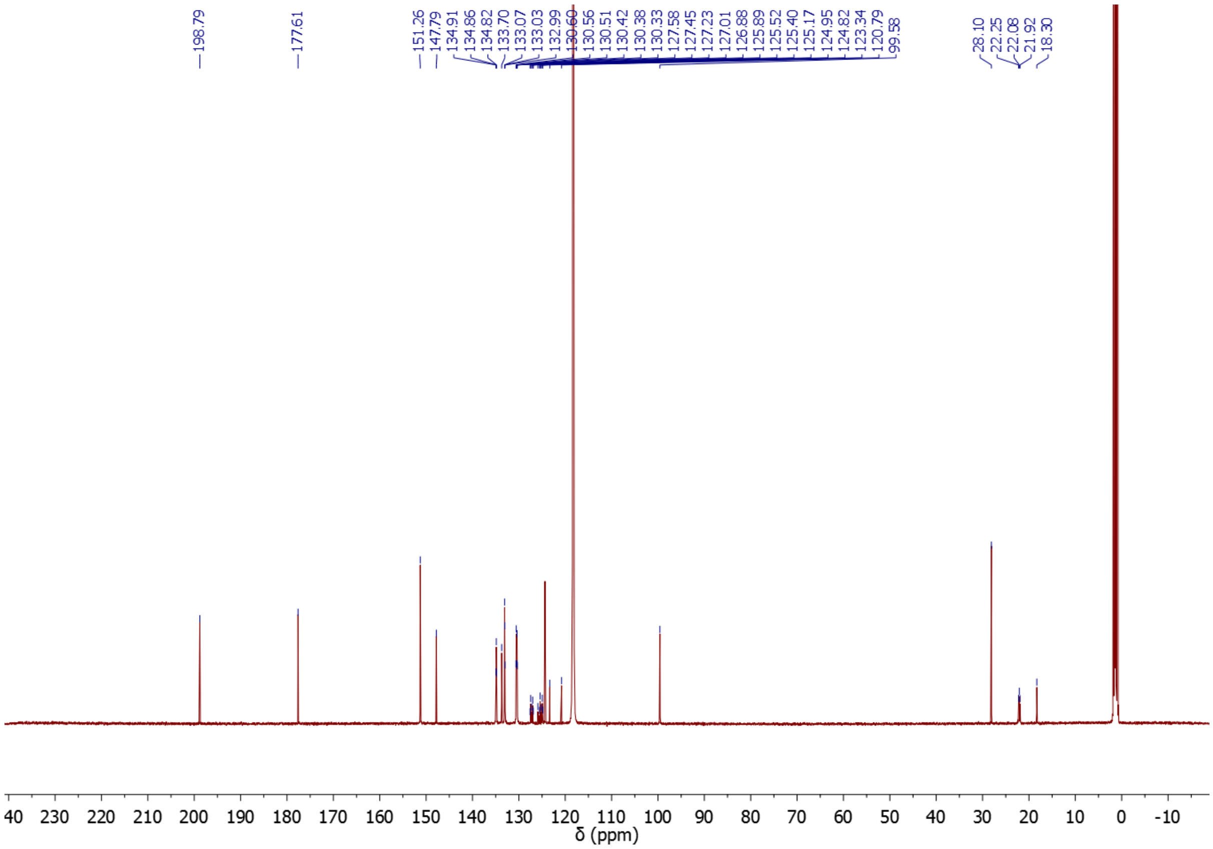

Figure S2. ^{13}C NMR (126 MHz, CD_3CN, 300 K) of **5**.

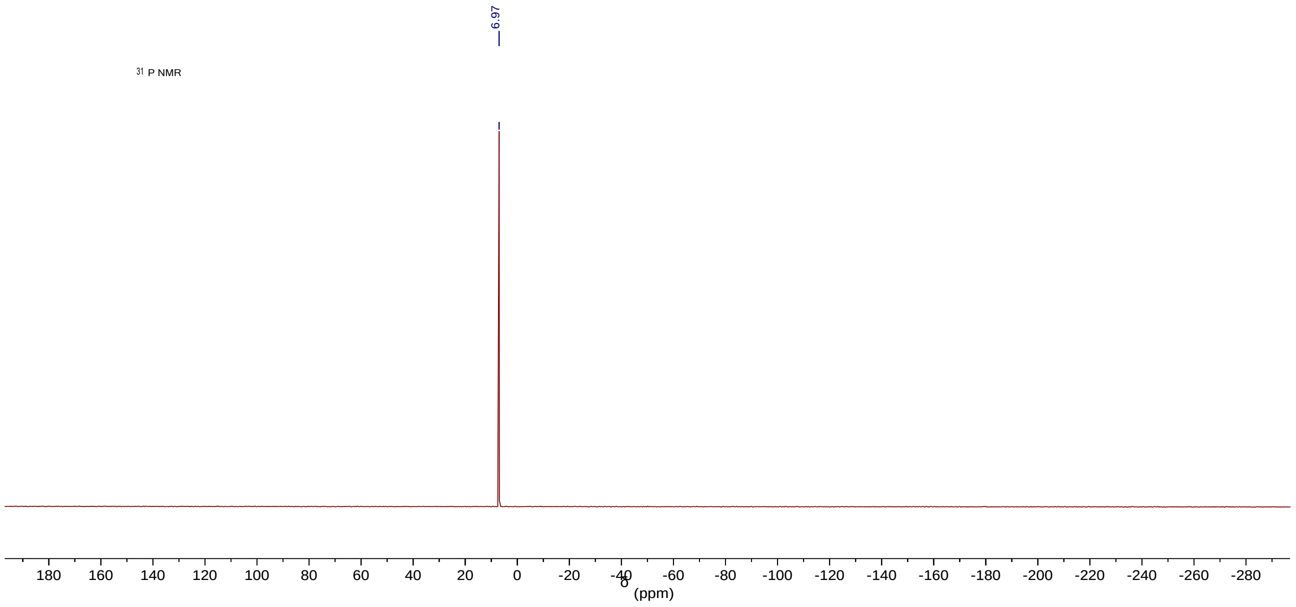

Figure S3. ^{31}P NMR (202 MHz, CD_3CN, 300 K) of **5**.

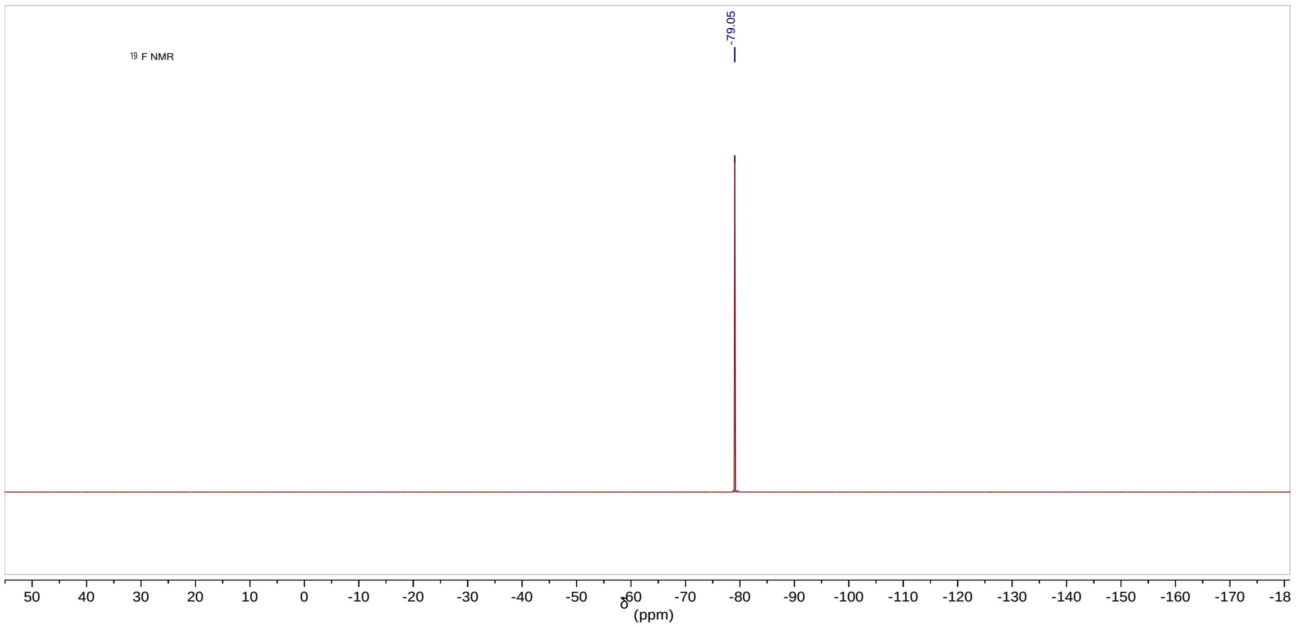

Figure S4. ^{19}F NMR (471 MHz, CD_3CN, 300 K) of **5**.

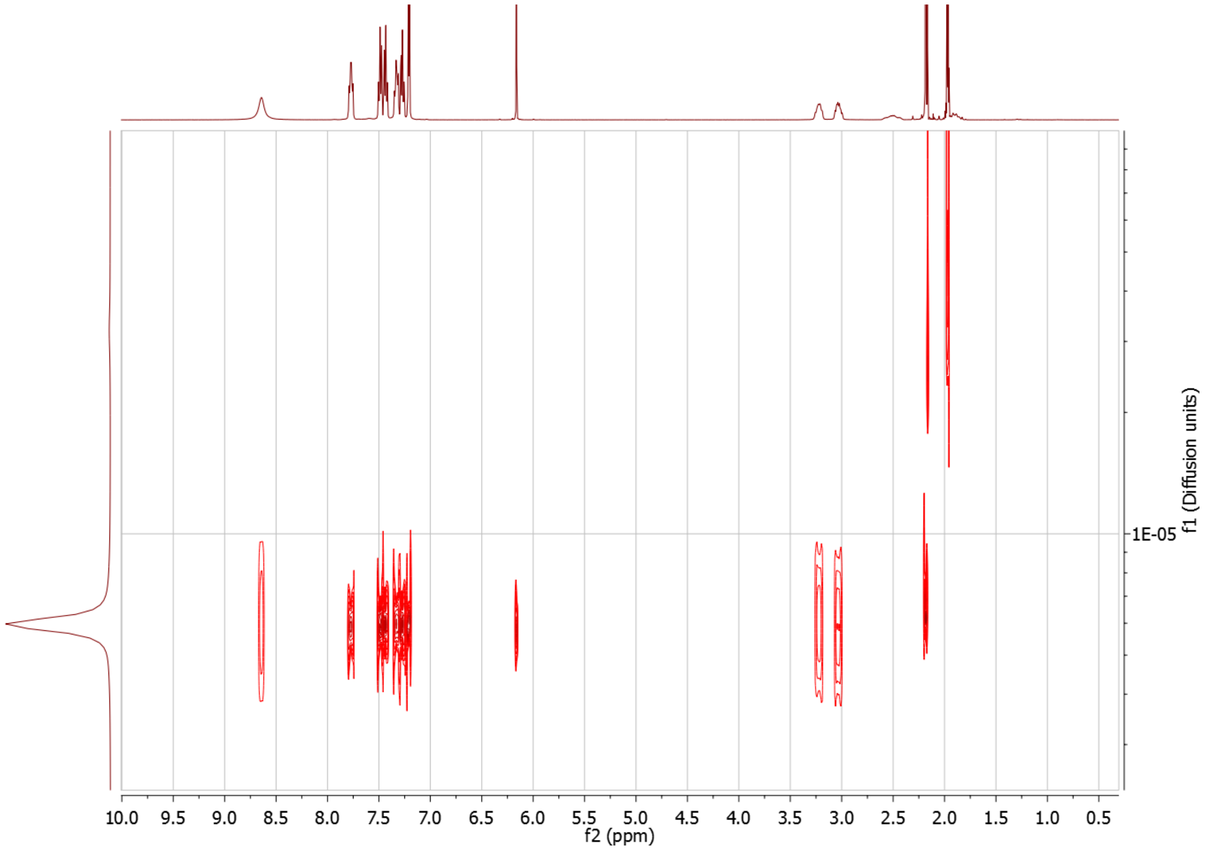

Figure S5. ^{1}H DOSY NMR (500 MHz, CD_3CN, 300 K) of **5**.

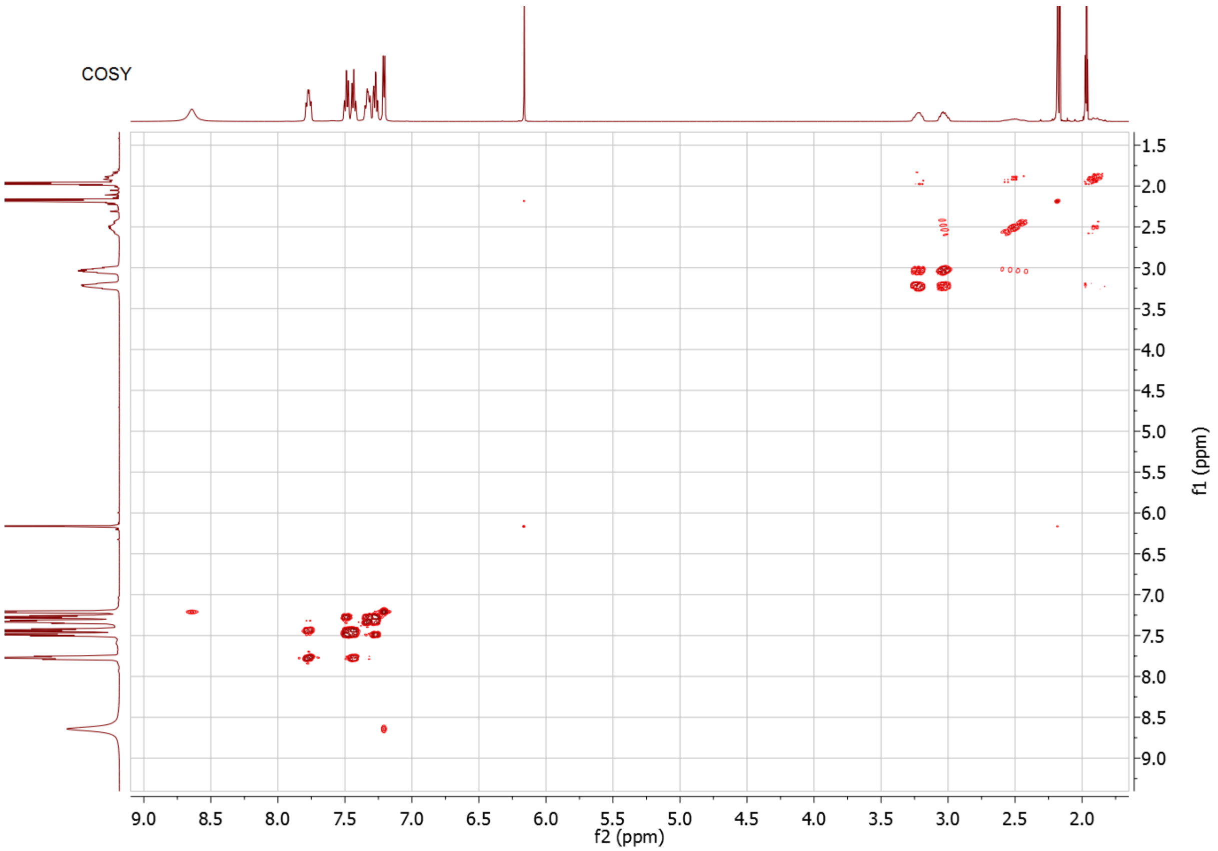

Figure S6. ^{1}H COSY NMR (500 MHz, CD_3CN, 300 K) of **5**.

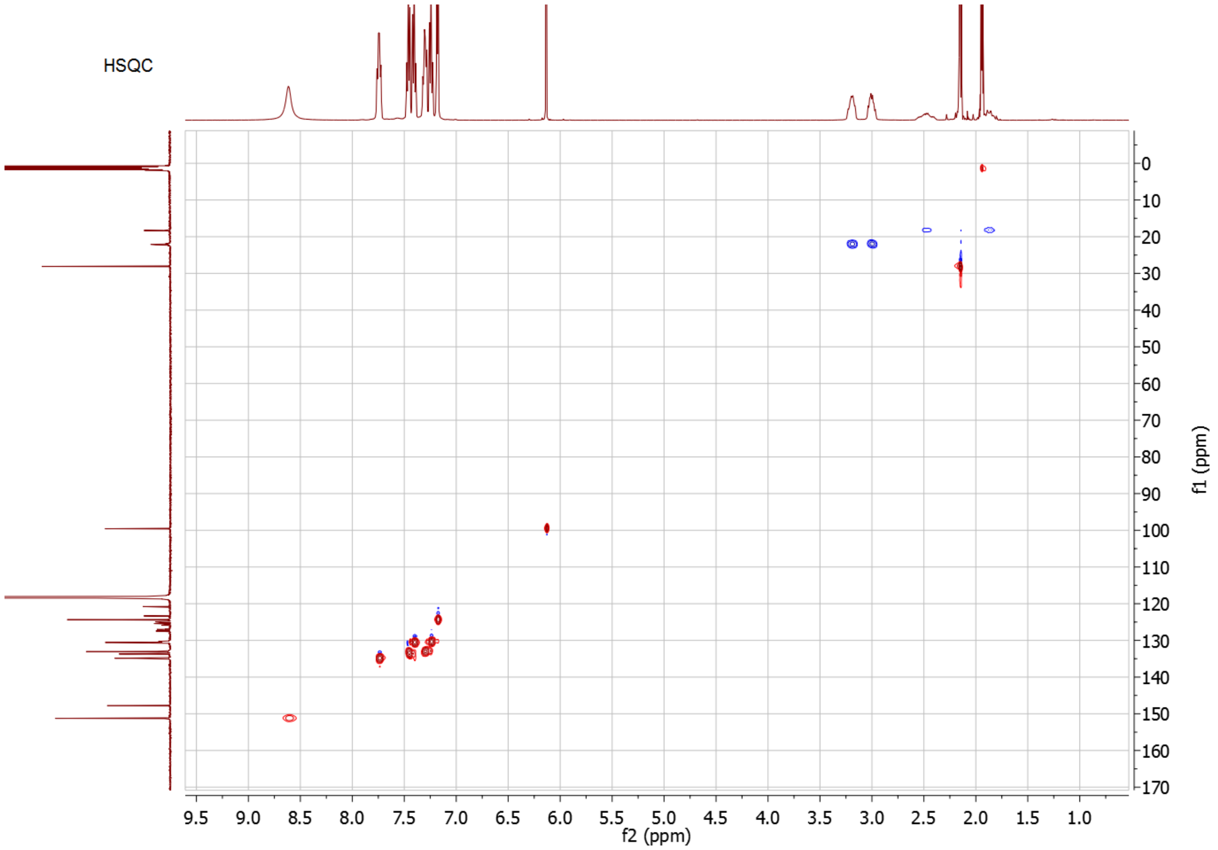

Figure S7. ^{1}H-^{13}C HSQC NMR (500 MHz, CD_3CN, 300 K) of **5**.

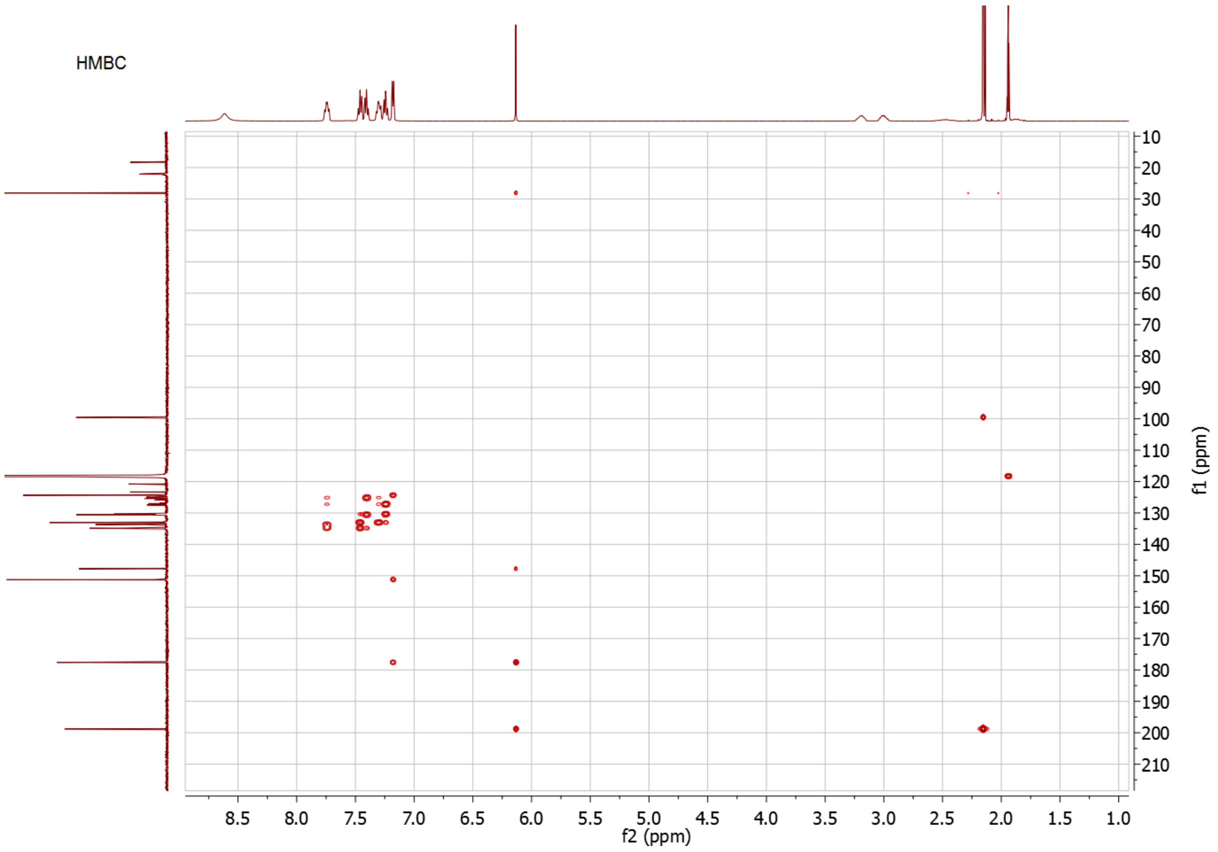

Figure S8. ^{1}H-^{13}C HMBC NMR (500 MHz, CD_3CN, 300 K) of **5**.

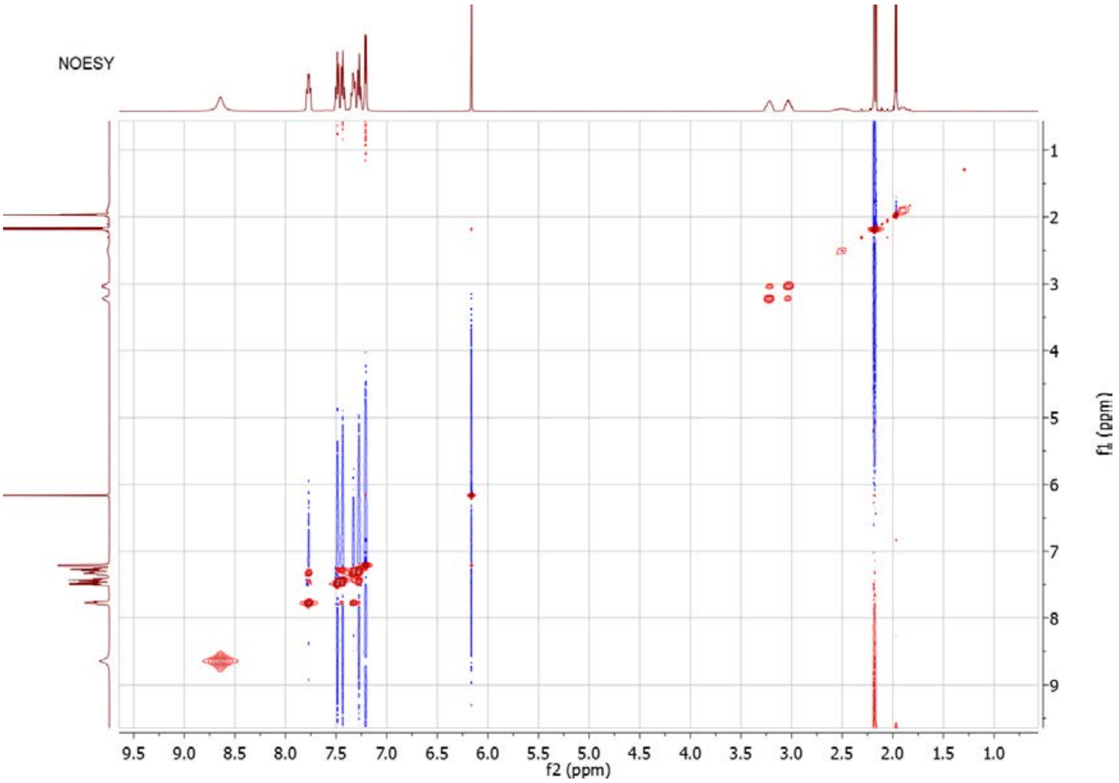

Figure S9. ^{1}H-^{13}C NOESY NMR (500 MHz, CD_3CN, 300 K) of **5**.

Mass Spectrometry

ESI TOF HRMS *m/z* for **FeL$_3$**: found 543.1051 [*M*+H]$^+$, 565.0865 [*M*+Na]$^+$; calculated for [$C_{27}H_{25}FeN_3O_6$]$^+$ 543.1088, [$C_{27}H_{24}FeN_3O_6Na$]$^+$ 565.0907.

ESI TOF HRMS *m/z* for **CrL$_3$**: found 539.1173 [*M*+H]$^+$, 561.0978 [*M*+Na]$^+$; calculated for [$C_{27}H_{25}CrN_3O_6$]$^+$ 539.1143, [$C_{27}H_{24}CrN_3O_6Na$]$^+$ 561.0963.

ESI TOF HRMS *m/z* for **AlL$_3$**: found 514.1544 [*M*+H]$^+$, calculated for [$C_{27}H_{25}AlN_3O_6$]$^+$ 540.1553.

ESI TOF HRMS *m/z* for compound **4**: Found 1026.7236 [M–3OTf $^-$]$^{3+}$, calculated for [$C_{138}H_{126}Cr_2F_9N_6O_{21}P_6Pd_3S_3$]$^{3+}$ 1026.7461.

ESI TOF HRMS *m/z* for compound **5**: Found 1010.0816 [M–3OTf $^-$]$^{3+}$, calculated for [$C_{138}H_{126}Al_2F_9N_6O_{21}P_6Pd_3S_3$]$^{3+}$ 1010.1068.

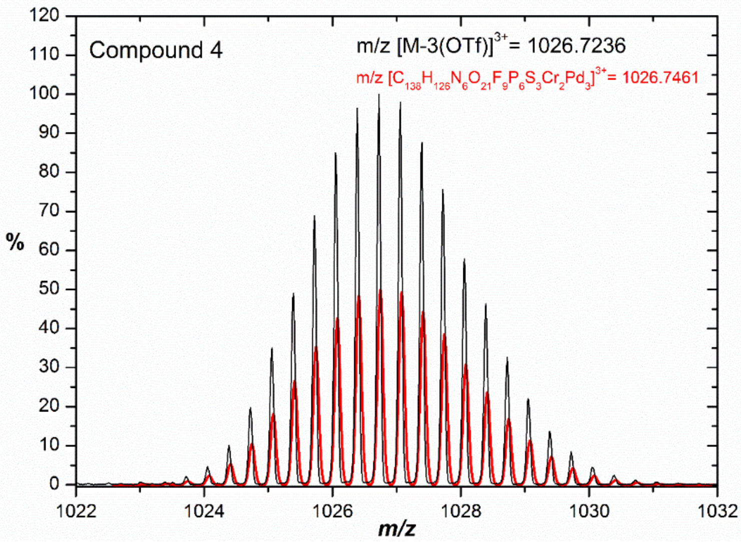

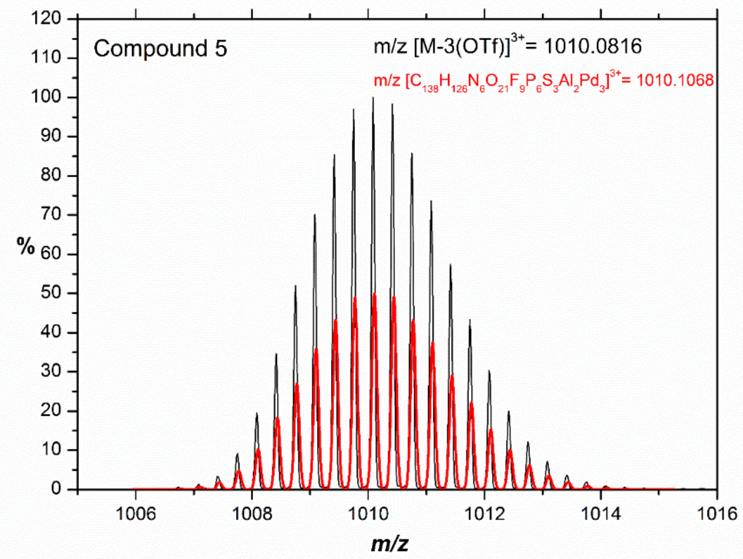

Figure S10. Partial ESI-MS of **4** (top) and **5** (bottom) showing isotopic distribution of the [**4**−3OTf$^-$]$^{3+}$ and [**5**−3OTf$^-$]$^{3+}$ charge state, respectively. Experimental (black) compared against theoretical (red).

EPR Spectroscopy

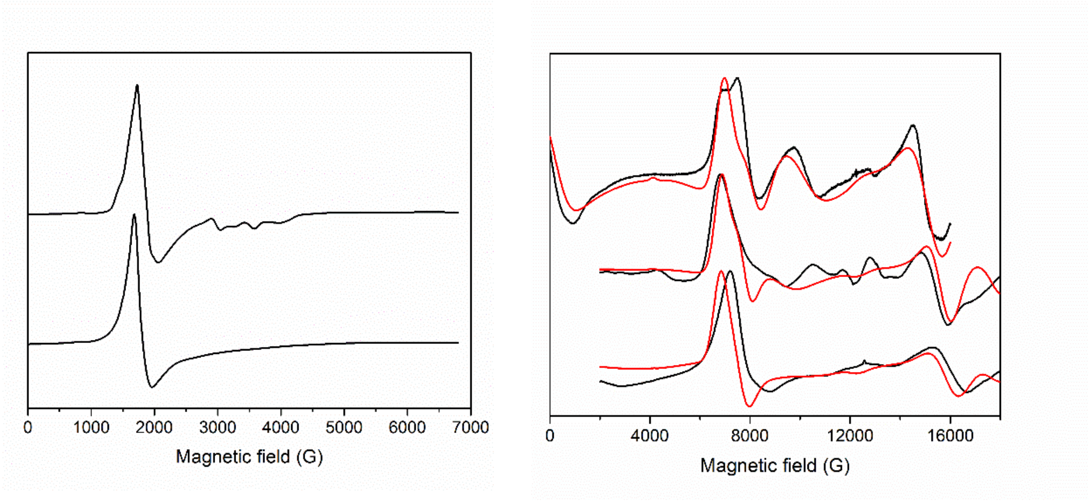

Figure S11. Left: X-band EPR spectra of powdered samples of **4** (top) and **3** (bottom) at 10 K. Right: Q-band EPR spectra of powdered samples of (from top to bottom) [CrL_3], **4** and **3** at 10 K (black), and calculated spectra (red) with D = -0.55 cm^{-1} ($|E/D|$ = 0.045) for [CrL_3], D = -0.61 cm-1 ($|E/D|$ = 0.036) for **4**, and D = -0.64 cm^{-1} ($|E/D|$ = 0.03) for **3** (with 400 G Gaussian linewidths and 5-10% D-strain). The X-band spectra are characteristic of a regime where $hv << |D|$, and hence are insensitive to $|D|$ above a threshold limit.

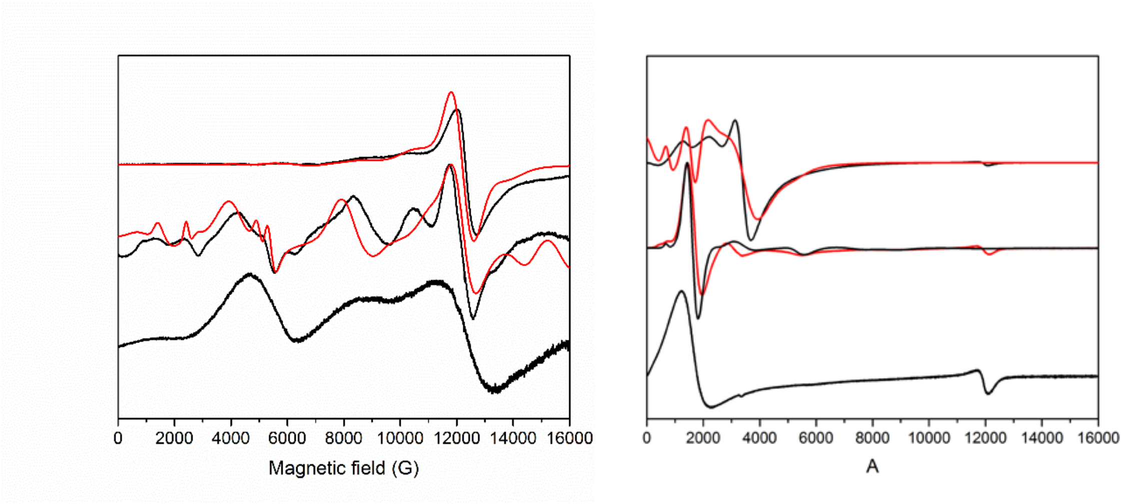

Figure S12. Q-band (left) and X-band (right) EPR spectra of powdered samples of (from top to bottom) [FeL_3], **2** and **1** at 5 K (black), and calculated spectra (red) with D = 0.08 cm^{-1} ($|E/D|$ = 1/3) for [FeL_3], and D = 0.20 cm^{-1} ($|E/D|$ = 1/3) for **2** (with 400 G Gaussian linewidths and 10-20% D-strain).

CASSCF methodology

CASSCF calculations were performed with MOLCAS 8.0,[1] where each Co^{II} site was treated independently and the other Co sites were replaced with the closed shell Zn^{II} ion. The active space consisted of seven electrons in the five 3d orbitals, where ten quartets and fourty doublets were considered both in the orbital optimisation (RASSCF) and the spin-orbit mixing (RASSI) procedures. The ZFS of the S = 3/2 ground state was extracted from the spin-orbit mixed states (SINGLE_ANISO). Basis sets from the ANO-RCC library of VTZP (Co), VDZP (coordinated N and Cl) and VDZ (all other atoms) quality were employed,[2] along with Cholesky decomposition of the two-electron integrals.

Table S1. CASSCF calculated ZFS of the $S = 3/2$ state of the three Co^{II} sites in **1**.

Site	Co1	Co2	Co3	Average
D (cm^{-1})	-11.8	-19.1	-11.9	-14
E (cm^{-1})	1.51	1.27	1.51	1.4
$\|E/D\|$	0.128	0.066	0.127	0.1

Powder X-RayDiffraction

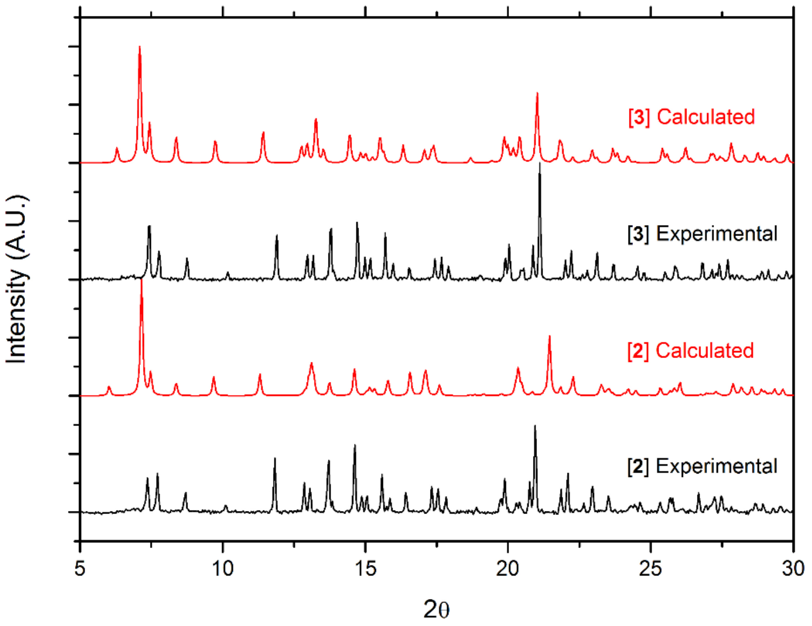

Figure S13. Powder X-ray diffraction overlay of calculated (red) and experimental (black) patterns of compounds **2** and **3**.

Inductively Coupled Plasma Optical Emission Spectroscopy (ICP-OES)

30 mg of crystalline sample of **1** was digested in 10 mL 20% *v*/*v* {HNO_3/HCl (1:3)} followed by a 10% dilution in 18.2 MΩ deionised water. The following range of multi element calibration standards for each element were prepared using single element 1000 mg/L standards (Fisher Scientific UK LTD Bishop Meadow Road, Loughborough, Leicestershire LE11 5RG), diluted with 18.2 MΩ deionised water: 0.01, 0.1, 1, 10 and 100 mg/L. With all the calibration lines the correlation coefficients for the linear regression were 0.999xx or better. After the analysis was completed the following wavelengths were selected for reporting results based on the shape of the peaks at that wavelength, background interferences, wavelength sensitivity and linearity of the calibration lines: Fe 259.939 nm and Co 228.616 nm. Experimental value of 19.496 ppm Fe and 31.669 ppm Co for **1** confirms its purity in terms of metal content as a Fe_2Co_3 cage.

References

1. F. Aquilante, J. Autschbach, R. K. Carlson, L. F. Chibotaru, M. G. Delcey, L. De Vico, I. Fdez. Galván, N. Ferré, L. M. Frutos, L. Gagliardi, M. Garavelli, A. Giussani, C. E. Hoyer, G. Li Manni, H. Lischka, D. Ma, P. Å. Malmqvist, T. Müller, A. Nenov, M. Olivucci, T. B. Pedersen, D. Peng, F. Plasser, B. Pritchard, M. Reiher, I. Rivalta, I. Schapiro, J. Segarra-Martí, M. Stenrup, D. G. Truhlar, L. Ungur, A. Valentini, S. Vancoillie, V. Veryazov, V. P. Vysotskiy, O. Weingart, F. Zapata and R. Lindh, *J. Comput. Chem.*, 2016, **37**, 506–541.

2. B. O. Roos, R. Lindh, P.-Å. Malmqvist, V. Veryazov and P.-O. Widmark, J. Phys. Chem. A, 2005, 109, 6575–6579.
